# CCG•CGG interruptions in high‐penetrance SCA8 families increase RAN translation and protein toxicity

**DOI:** 10.15252/emmm.202114095

**Published:** 2021-10-11

**Authors:** Barbara A Perez, Hannah K Shorrock, Monica Banez‐Coronel, Tao Zu, Lisa EL Romano, Lauren A Laboissonniere, Tammy Reid, Yoshio Ikeda, Kaalak Reddy, Christopher M Gomez, Thomas Bird, Tetsuo Ashizawa, Lawrence J Schut, Alfredo Brusco, J Andrew Berglund, Lis F Hasholt, Jorgen E Nielsen, SH Subramony, Laura PW Ranum

**Affiliations:** ^1^ Center for NeuroGenetics University of Florida Gainesville FL USA; ^2^ Department of Molecular Genetics and Microbiology University of Florida Gainesville FL USA; ^3^ Department of Neurology Gunma University Maebashi Japan; ^4^ RNA Institute University at Albany–SUNY Albany NY USA; ^5^ Department of Neurology University of Chicago Chicago IL USA; ^6^ Department of Neurology University of Washington Seattle WA USA; ^7^ Geriatrics Research Section VA Puget Sound Health Care System Seattle WA USA; ^8^ Department of Neurology Houston Methodist Research Institute Houston TX USA; ^9^ Department of Neurology University of Minnesota Minneapolis MN USA; ^10^ Department of Medical Sciences University of Torino Torino Italy; ^11^ Medical Genetics Units “Città della Salute e della Scienza” University Hospital Torino Italy; ^12^ Institute of Cellular and Molecular Medicine University of Copenhagen Copenhagen Denmark; ^13^ Department of Neurology Rigshospitalet University of Copenhagen Copenhagen Denmark; ^14^ McKnight Brain Institute University of Florida Gainesville FL USA; ^15^ Genetics Institute University of Florida Gainesville FL USA

**Keywords:** *cis*‐modifier, RAN translation, reduced penetrance, sequence interruptions, spinocerebellar ataxia type 8, Genetics, Gene Therapy & Genetic Disease, Neuroscience

## Abstract

Spinocerebellar ataxia type 8 (SCA8), a dominantly inherited neurodegenerative disorder caused by a CTG•CAG expansion, is unusual because most individuals that carry the mutation do not develop ataxia. To understand the variable penetrance of SCA8, we studied the molecular differences between highly penetrant families and more common sporadic cases (82%) using a large cohort of SCA8 families (*n* = 77). We show that repeat expansion mutations from individuals with multiple affected family members have CCG•CGG interruptions at a higher frequency than sporadic SCA8 cases and that the number of CCG•CGG interruptions correlates with age at onset. At the molecular level, CCG•CGG interruptions increase RNA hairpin stability, and in cell culture experiments, increase p‐eIF2α and polyAla and polySer RAN protein levels. Additionally, CCG•CGG interruptions, which encode arginine interruptions in the polyGln frame, increase toxicity of the resulting proteins. In summary, SCA8 CCG•CGG interruptions increase polyAla and polySer RAN protein levels, polyGln protein toxicity, and disease penetrance and provide novel insight into the molecular differences between SCA8 families with high vs. low disease penetrance.

## Graphical abstract

This study shows CCG•CGG interruptions within the *ATXN8OS/ATXN8* CTG•CAG repeat are an important genetic modifier of disease penetrance in spinocerebellar ataxia type 8 (SCA8).

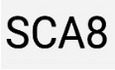

**The paper explained**

**Problem**

Spinocerebellar ataxia type 8 (SCA8) is unusual because most individuals that carry the CTG•CAG repeat expansion mutation do not develop ataxia.

**Results**

Using a large SCA8 patient cohort, we demonstrate that CCG•CGG interruptions in the SCA8 CTG•CAG repeat expansion are associated with increased penetrance of disease and account for 37% of the variation in age of onset in SCA8 patients with interrupted alleles. At the molecular level, CCG•CGG‐interrupted CTG•CAG repeat expansions are more toxic to cells than pure CTG•CAG repeat expansions and lead to increased steady‐state levels of polyAla and polySer proteins produced through repeat‐associated non‐AUG (RAN) translation.

**Impact**

Our study shows the number of CCG•CGG interruptions is correlated with increased disease penetrance and decreased age of onset in SCA8 patients. CGG‐interrupted *ATXN8* CAG expansions are more toxic to cells than pure repeats. These interruptions increase RNA stability, activate the integrated stress response, and increase polyAla and polySer RAN protein levels and the toxicity of the resulting arginine‐interrupted polyGln proteins. These data provide novel molecular insight into the variable penetrance of SCA8 and the risk of developing the disease.

## Introduction

Spinocerebellar ataxia type 8 (SCA8) is a microsatellite expansion disorder caused by a bidirectionally transcribed CTG•CAG repeat expansion mutation within the *ATXN8OS*/*ATXN8* genes (Koob *et al*, 1999; Moseley *et al*, 2006). This slowly progressive cerebellar ataxia is typically characterized by ataxia, spasticity, dysarthria, and nystagmus; however, extra‐cerebellar features including psychiatric disturbances and developmental delays have been reported (Day *et al*, 2000; Juvonen *et al*, 2000; Stone *et al*, 2001; Lilja *et al*, 2005; Koutsis *et al*, 2012; Kim *et al*, 2013; Cleary *et al*, 2021; Zhou *et al*, 2019). Although SCA8 is caused by a dominantly inherited mutation, patients frequently have no family history of ataxia and are referred to as sporadic cases. Despite the negative family history, repeat expansion mutations are almost always found in asymptomatic relatives of these sporadic cases (Koob *et al*, 1999; Moseley *et al*, 2000b; Worth *et al*, 2000; Ikeda *et al*, 2004). Additionally, the age of onset and clinical features of the disease vary widely among affected individuals, with onset reported from birth to 73 years of age (Koob *et al*, 1999; Day *et al*, 2000; Ikeda *et al*, 2000, 2004; Silveira *et al*, 2000; Felling & Barron, 2005; Lilja *et al*, 2005; Kim *et al*, 2013; Samukawa *et al*, 2019).

Repeat‐associated non‐AUG (RAN) proteins, which were first discovered in SCA8 and DM1 (Zu *et al*, 2011), have now been described in 11 microsatellite expansion disorders (Zu *et al*, 2011, 2017; Mori *et al*, 2013; Todd *et al*, 2013; Banez‐Coronel *et al*, 2015; Buijsen *et al*, 2016; Ishiguro *et al*, 2017; Banez‐Coronel & Ranum, 2019; Goodman & Bonini, 2019; McEachin *et al*, 2020). RAN translation is a process in which transcripts containing repeat expansions express proteins in multiple reading frames without the requirement of AUG‐ or AUG‐like close‐cognate initiation codons (Zu *et al*, 2011, 2018; Cleary *et al*, 2018; Banez‐Coronel & Ranum, 2019; Nguyen *et al*, 2019). The presence of RAN and ATG‐initiated expansion proteins has been previously reported in human SCA8 autopsy brains and SCA8‐BAC transgenic mice (Moseley *et al*, 2006; Zu *et al*, 2011; Ayhan *et al*, 2018). Both ATG‐initiated polyglutamine (polyGln) and RAN poly‐Alanine (polyAla) proteins are found in Purkinje cells (Moseley *et al*, 2006; Zu *et al*, 2011) and polyGln and RAN poly‐Serine (polySer) proteins are detected in the hippocampus, pons, and frontal cortex (Ayhan *et al*, 2018). Additionally, polySer aggregates accumulate in the cerebellar white matter and brainstem nuclei where they are associated with demyelination, axonal degeneration, increased astrogliosis, and a reduction in the number of mature oligodendrocytes (Ayhan *et al*, 2018).

In contrast to other SCAs, SCA8 is unusual in that there is markedly reduced penetrance (Koob *et al*, 1999; Stevanin *et al*, 2000; Worth *et al*, 2000; Ikeda *et al*, 2004), suggesting that genetic and/or environmental modifiers affect the onset and penetrance of SCA8. One potential genetic modifier that may affect disease penetrance in SCA8 is the presence of repeat interruptions. The presence of CCG, CTA, CTC, CCA, and CTT interruptions in the CTG repeat expansion in SCA8 have previously been reported (Moseley *et al*, 2000b; Hu *et al*, 2017). These interruptions can vary in number, configuration, and position within the repeat tract. Interestingly, one to four CCG interruptions were detected in multiple configurations among affected members of a large, highly penetrant SCA8 family (MN‐A) and the number of interruptions often increases when passed from one generation to the next (Moseley *et al*, 2000b).

Repeat interruptions have been reported to have different modifying effects in a number of other microsatellite expansion disorders. For example in SCA1, SCA2, and fragile‐X syndrome (FXS), sequence interruptions appear to stabilize unexpanded repeat tracts, and the loss of interruptions predisposes repeat tracts to expand above the pathogenic threshold (Chung *et al*, 1993; Kunst & Warren, 1994; Imbert *et al*, 1996; Pulst *et al*, 1996; Sanpei *et al*, 1996; Gunter *et al*, 1998). In other cases, interruptions are found on expanded alleles and are associated with changes in disease presentation. For example, CAA interruptions on expanded alleles are associated with later ages of onset in SCA2 (Sobczak & Krzyzosiak, 2005) and Huntington disease (Genetic Modifiers of Huntington's Disease, 2019; Wright *et al*, 2019). In SCA10, patients with ATCCT interruptions are prone to seizures (McFarland *et al*, 2014), and in DM1, CCG and GGC interruptions are found in patients with peripheral neuropathy (Braida *et al*, 2010), but the molecular basis for these effects is unclear.

Here, we show that CCG•CGG interruptions are preferentially found on expanded alleles in SCA8 families with increased disease penetrance and that age of onset is inversely correlated with the number of interruptions and not repeat length. Molecular studies in cell culture show CCG•CGG interruptions increase p‐eIF2α, polyAla, and polySer RAN protein levels and the toxicity of the resulting arginine‐interrupted polyGln expansion proteins. Our demonstration that CCG•CGG interruptions increase RAN protein levels and polyGln protein toxicity and are found in families with increased disease penetrance provides novel molecular insight into the variable penetrance and risk of developing SCA8.

## Results

### Most SCA8 patients have no family history of ataxia

**Figure 1:**
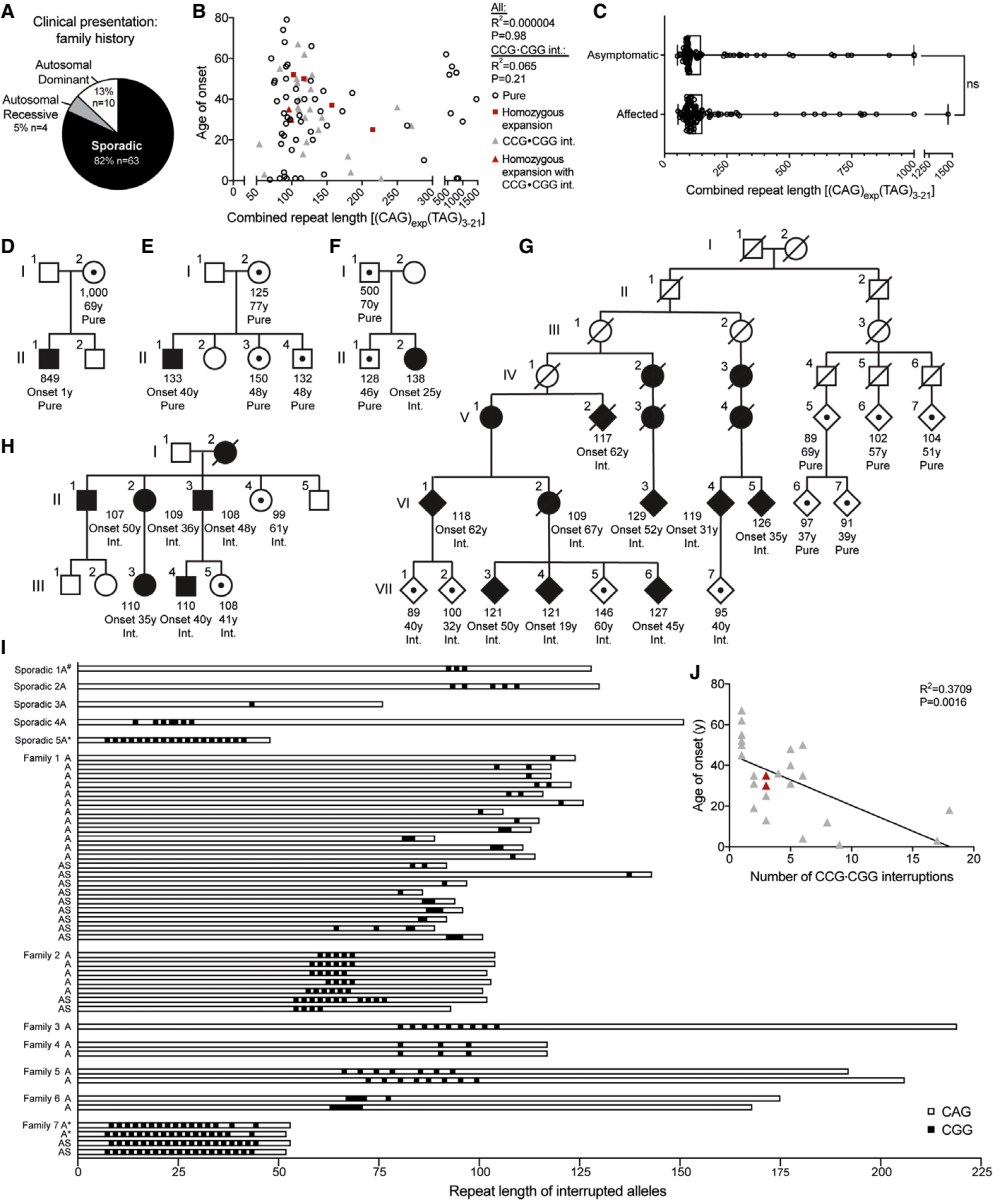
SCA8 alleles with CCG•CGG interruptions are found in families with high disease penetrance ASummary of disease history in the 77 SCA8 families in our cohort.BNo correlation between length of combined repeats and age of onset in SCA8 patients, *n* = 85, *P* = 0.9847 or in the subset of SCA8 patients with CCG•CGG interruptions (Int.), *n* = 26, *P* = 0.2096, linear regression analyses. Open circles indicate SCA8 patients with pure CAG repeat expansions. Red squares show average expansion size for individuals with two expanded alleles, individual allele repeat lengths: 137/177,110/320, 104/130, 96/109. Red triangles show average expansion size for individuals with two expanded alleles and CCG•CGG interruptions: 84/114, 92/100. Gray triangles indicate individuals with CCG•CGG interruptions.CAllele length distribution of affected (*n* = 111) and asymptomatic (*n* = 88) expansion carriers; box: 25^th^ to 75^th^ percentile; whiskers: minimum to maximum value; *P* = 0.0672, Mann–Whitney test.D–HSCA8 family pedigrees: Squares represent males, circles represent females, and diamonds mask gender. Filled symbols represent affected individuals, symbols with dot represent asymptomatic expansion carriers, open symbols represent individuals with non‐expanded alleles, and diagonal line indicates a deceased individual. Combined repeat number, age (y—years) at onset (Onset) or age still asymptomatic, and interruption status (Pure or Int. [CCG•CGG‐interrupted]) are noted below the symbols. (G) Abbreviated pedigree, for additional details, see Koob *et al* (1999).ISCA8 allele configurations in the CAG direction as determined by sequencing. Family or individual and affected status indicated on left: Sporadic 1—Fig 1F indicated by ^#^, Family 1—Fig 1G, Family 2—Fig 1H; A—affected, AS—Asymptomatic; CGG interruptions represented by black boxes. Sequences used in Fig 5C are indicated by *. See Appendix Table S1 for allele configurations.JAge of onset correlates with the number of CCG•CGG interruptions, *n* = 24, *P* = 0.0016, linear regression analyses. Gray triangles indicate individuals with CCG•CGG interruptions. Red triangles indicate the average expansion size for individuals with two expanded alleles and CCG•CGG interruptions, and individual allele repeat lengths are as follows: 84/114, 92/100. Data information: (I, J) Individuals identified as having CCG•CGG interruptions by restriction digest are not included.

To investigate the effects of sequence interruptions in SCA8, we performed a detailed genetic evaluation of expanded SCA8 alleles from a large cohort of SCA8 families (*n* = 77) including 199 expansion carriers (*n* = 111 affected, *n* = 88 asymptomatic). Disease onset ranged from birth to 79 years with an average age of onset of 33.7 years (Table 1). Although the mutation is transmitted in an autosomal‐dominant pattern, surprisingly 82% (63/77) of these families had sporadic ataxia with no family history of disease, 5% (4/77) had family histories that appeared recessive, and only 13% (10/77) showed the expected autosomal‐dominant inheritance pattern (Fig 1A). Interestingly, four of the sporadic and two familial cases are homozygous and have two expanded alleles. These data and previous reports of expansion alleles in unaffected family members and in the general population (Moseley *et al*, 2000a; Stevanin *et al*, 2000; Worth *et al*, 2000; Cellini *et al*, 2001; Ikeda *et al*, 2004; Zeman *et al*, 2004) highlight the need to understand the molecular basis of the variable penetrance found in SCA8.

**Table 1:** Repeat length is not a reliable predictor of SCA8 disease status.

	Affected *n* = 111	Asymptomatic *n* = 88	*P*‐value
Avg age onset (years)	33.7 ± 19.7	–	
Females, *n* (%)	57 (51.35)	49 (55.68)	ns
Males, *n* (%)	54 (48.65)	39 (44.32)	–
Combined repeat #			
Mean	189 ± 219.9	188.5 ± 211.1	–
Median	113	98	ns
Maximum	1,455	1,000	–
Minimum	54	52	–

Characteristics of participants are presented as mean ± SD, number, and percentage of affected or asymptomatic individuals (%). To determine group effect, Fisher's exact test was used for categorical variables and Mann–Whitney test for non‐parametric continuous variables. Average age of onset (Avg age onset) *n* = 85. ns: not significant.

### SCA8 repeat length does not correlate with age of onset or predict disease status

Consistent with previous reports (Juvonen *et al*, 2000; Ikeda *et al*, 2004; Zeman *et al*, 2004; Cleary *et al*, 2021), we found: (i) no correlation in the number of SCA8 repeats and age of onset (Fig 1B); (ii) no significant difference in repeat length between affected patients (median: 113 repeats) and asymptomatic expansion carriers (median: 98 repeats; *P* = 0.0672; Table 1); and (iii) a wide and overlapping range of repeat lengths in affected (54–1,455) and asymptomatic expansion carriers (52–1,000; Fig 1C; Table 1). The lack of correlation between repeat length and disease status is often seen in individual SCA8 families. For example, in Fig 1D, individual I‐2 carries an expansion of 1,000 repeats yet remains asymptomatic, while her son with 849 repeats presented with disease at 1 year of age. Similarly, in Fig 1E, individual II‐1 presented with disease at age 40 with 133 combined repeats while his mother and two siblings, who carry SCA8 expansions of similar lengths, remain asymptomatic. Taken together, these data provide additional evidence that repeat length is not a reliable predictor of disease or age of onset and suggest other genetic or environmental modifiers contribute to the variable penetrance of SCA8.

A potential genetic modifier of SCA8 is the presence of interruptions within the CAG repeat expansion. In Fig 1F, a 25‐year‐old female (II‐2), with no family history of SCA8, has an expansion mutation containing three *de novo* CGG interruptions [(CAG)_91_(CAGCGG)_3_(CAG)_31_(TAG)_10_]. These interruptions were not present in her asymptomatic 70‐year‐old father (I‐1; confirmed pure by MspA1I digest) or 46‐year‐old brother (II‐1; (CAG)_118_(TAG)_10_; Fig 1F). The observation that the only affected individual in this family has CCG•CGG interruptions, combined with the previously reported CCG•CGG interruptions in affected members of an unusually large SCA8 kindred (Moseley *et al*, 2000b), suggested to us that CCG•CGG interruptions are associated with increased disease penetrance.

### CCG•CGG interruptions increase disease penetrance and inversely correlate with age of onset

**Figure EV1:**
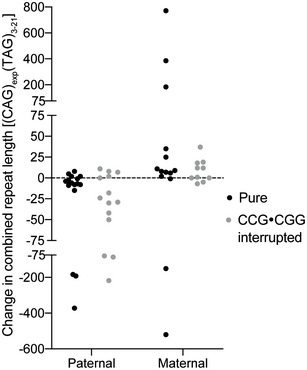
CCG•CGG interruptions do not affect inheritance patterns of SCA8 CTG•CAG repeat expansions No differences in repeat length changes for paternal or maternal transmissions of pure versus CCG•CGG‐interrupted alleles. Paternal transmission of pure alleles: *n* = 16, CCG•CGG‐interrupted alleles: *n* = 14. Maternal transmission of pure alleles: *n* = 14, CCG•CGG‐interrupted alleles: *n* = 10. Individuals carrying two expanded alleles were included if sequencing could determine transmitted allele. No significant differences in repeat length changes were found for pure versus interrupted transmissions of paternal (*P* = 0.5314) or maternal alleles (*P* = 0.5748) using Mann–Whitney test. Dashed line represents no change in repeat length.

To better understand the effects of CCG•CGG interruptions on disease penetrance, we compared the sequences of SCA8 expansion alleles in families with high (≥ 3 affected) versus low disease penetrance. The seven‐generation MN‐A family (Koob *et al*, 1999; Day *et al*, 2000), the largest SCA8 family reported to date, has a much higher disease penetrance than most SCA8 families (Ikeda *et al*, 2004), and CCG•CGG interruptions were previously reported in all affected individuals (Moseley *et al*, 2000b). Additional analyses of this family show CCG•CGG interruptions are found in the high penetrance branch but not a newly identified low‐penetrance branch of this family. The left family branch shows an autosomal‐dominant inheritance pattern (onset 19–74 years; Fig 1G) while members of the extended right branch have pure CTG•CAG expansions and no affected individuals (Fig 1G). In a second newly identified multigenerational family, all six affected individuals (onset 35–50 years; Fig 1H) have CCG•CGG interruptions. These interruptions were also identified in individual II‐4 who was not affected at the time of examination but subsequently showed signs of ataxia and in individual III‐5 who was asymptomatic at age 41 (Fig 1H). CCG•CGG interruptions were found at a higher frequency in families with multiple affected individuals: 100% (5/5) of families with three or more affected individuals, 28.6% (2/7) of families with two affected individuals, and 13.9% (5/36) of sporadic cases. Overall, CCG•CGG interruptions were found at a higher frequency in SCA8 families with two or more affected members compared with sporadic cases (*n* = 48; *P* = 0.0047; Table 2) and among affected individuals compared with asymptomatic carriers (*n* = 132; *P* = 0.0299; Table 2; Appendix Tables S1 and S2). While the position, configuration and number of CCG•CGG interruptions varies widely among SCA8 families (Fig 1I, Appendix Table S1), the number of CCG•CGG interruptions is inversely correlated with and accounts for 37% of the variation in age of onset (*R* ^2^ = 0.3709; *P* = 0.0016; Fig 1J). We saw no significant difference in changes in repeat length on paternal or maternal transmission of pure versus CCG•CGG‐interrupted alleles (paternal transmission *P* = 0.5314, maternal transmission *P* = 0.5748; Fig EV1).

**Table 2:** CGG interruptions are associated with increased disease penetrance in SCA8.

Families	Pure	CGG interrupted	*P*‐value
Apparent sporadic	31 (86%)	5 (14%)	0.0047
2+ affected	5 (42%)	7 (58%)	

Individuals	Pure	CGG interrupted	*P*‐value
Affected	41 (55%)	33 (45%)	0.0299
Asymptomatic	43 (74%)	15 (26%)	

*P*‐values were calculated using Fisher's exact test to assess the relationship between disease penetrance and CGG interruptions (*n* = 48 families; *n* = 132 expansion carriers) See Appendix Tables S1 and S2 for allele configurations. One family with CGG‐interrupted alleles (Fig 1I Family 4) contained a single GAG between the CAG expansion and the polymorphic TAG tract. An additional *n* = 10 families, representing *n* = 19 expansion carriers, were sequenced and found to carry different interruptions. The interruptions in these families included CTG, TAG, TGG, AAG, GAG, TAC, CCG (in the CAG direction) were not found in combination with CGG interruptions and were not included above; see Appendix Table S3 for all allele configurations.

Taken together, these data demonstrate that CCG•CGG interruptions increase disease penetrance and that the number of interruptions, and not repeat length, is inversely correlated with age at onset in SCA8.

### CCG•CGG interruptions increase the toxicity of SCA8 CTG•CAG repeat expansions

**Figure 2:**
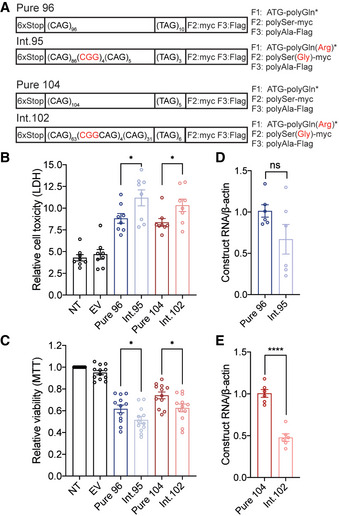
Clustered and interspersed CCG•CGG interruptions increase toxicity of CTG•CAG expansions ASchematic diagram of constructs used to express patient‐derived pure and interrupted SCA8 repeat tracts with predicted protein products and C‐terminal epitope tags. * Due to TAG encoded stop codons, polyGln proteins do not express an epitope tag. CGG interruptions and the encoded amino acid interruptions are indicated in red.B, CCell death measured by lactase dehydrogenase (LDH) (B) and cell viability measured by 3‐(4,5‐dimethyl‐thiazol‐. 2‐yl)‐2,5‐diphenyl tetrazolium bromide (MTT) (C) in T98 cells 42 hrs post‐transfection of pure and interrupted SCA8 repeat tracts; LDH *n* = 8, MTT *n* = 12, *n* = independent experiments, * *P* < 0.05, NT: not transfected, EV: empty vector; unpaired *t*‐test; mean ± SEM.D, ERT–qPCR showing transcript levels of Pure 96 and Int.95 (D, *n* = 6; *P* = 0.0187), and Pure 104 and Int.102 (E, *n* = 6; **** *P* < 0.0001) repeats; *n* = independent experiments; unpaired *t*‐test; mean ± SEM.

To better understand the molecular effects of interrupted alleles, we tested whether constructs containing CCG•CGG interruptions are more toxic to cells than pure repeat expansion constructs. T98 glial cells were transfected with length‐matched constructs containing pure or interrupted expansions cloned from patient DNA and expressed in the CAG direction (Fig 2A). Interrupted expansions were cloned from individuals from the high‐penetrance multigeneration families shown in Fig 1G (Int.95) and Fig 1H (Int.102). Int.95 contains an overall CAG repeat length of 95 with four consecutive CGG interruptions near the 3′ end, followed by three TAGs [(CAG)_86_(CGG)_4_(CAG)_5_(TAG)_3_]. Int.102 contains four mixed CAGCGG interruptions in the middle of the CAG repeat for a total of 102 interrupted CAGs followed by six TAGs [(CAG)_63_(CGGCAG)_4_(CAG)_31_(TAG)_6_] (Fig 2A). Cells expressing these interrupted constructs showed increased death (26.9%, *P* < 0.05 – Int.95 vs Pure 96; 23.5%, *P* < 0.05 – Int.102 vs Pure 104; Fig 2B) and decreased viability (16.5%, *P* < 0.05 – Int.95 vs Pure 96; 15.6%, *P* < 0.05 – Int.102 vs Pure 104; Fig 2C) compared with length‐matched uninterrupted repeats. These effects cannot be explained by differences in RNA levels which did not differ in Pure 96 versus Int.95‐transfected cells and were lower in Int.102 vs Pure 104‐transfected cells (Fig 2D and E). Taken together, these data indicate that CGG interruptions increase the toxicity of CAG repeats independent of RNA levels.

### Arginine‐encoding CGG interruptions increase toxicity of polyGln proteins

**Figure 3:**
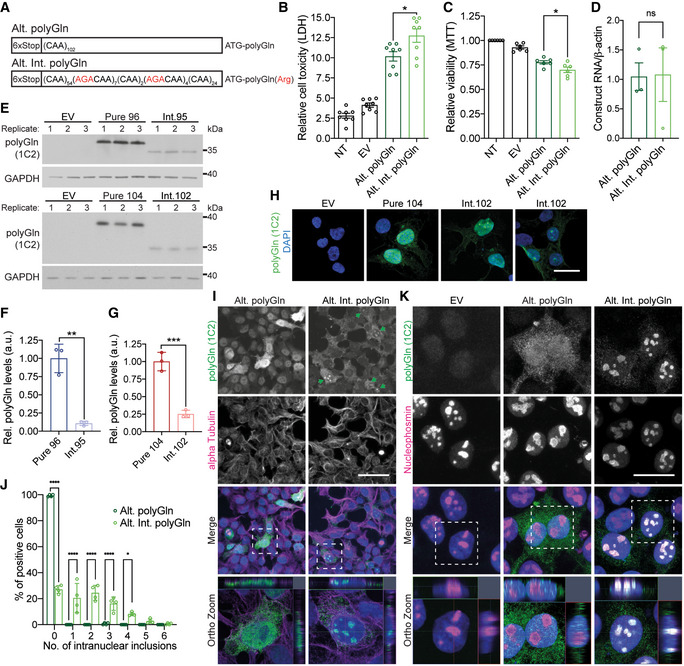
Arginine interruptions increase toxicity of *ATXN8* polyGln proteins AAlternative‐codon constructs for pure and interrupted polyGln assays.B, CCell death (B; LDH, *n* = 8) and viability (C; MTT, *n* = 6) assays in T98 cells 42 h post‐transfection. NT: not transfected; EV: empty vector; *n* = independent experiments; * *P* < 0.05; unpaired *t*‐test; mean ± SEM.DRT–qPCR of Alt. polyGln and Alt. Int. polyGln construct transcript levels, *n* = 3 independent experiments; *P* = 0.9516, ns: not significant, unpaired *t*‐test, mean ± SEM.E–GProtein blots (E) and quantification (F, G) of polyGln proteins expressed in transfected HEK293T cells with interrupted or pure polyGln repeats; EV: empty vector; *n* = 3 transfections, ** *P* < 0.01, *** *P* < 0.001, unpaired *t*‐test, mean ± SD.HImmunofluorescence of polyGln expressed from Pure 104 and Int.102 constructs in HEK293T cells; scale bar: 20 μm; EV: empty vector.I, JImmunofluorescence (I) and quantification (J) of intranuclear inclusions for polyGln expressed from Alt. polyGln and Alt. Int. polyGln constructs in HEK293T cells; scale bar: 50 μm; green arrowheads indicate cells positive for Alt. Int. polyGln intranuclear inclusions; dashed white box in merge panels indicates the region shown in the orthogonal (Ortho) zoomed images which show cross section and localization of diffuse polyGln staining and nuclear polyGln(Arg) inclusions with alpha‐tubulin as a cytoplasmic marker; *n* = 4 experiments with *n* > 55 cells per experiment, *P < 0.05, ****P < 0.0001, two‐way ANOVA, data presented as mean percentage of polyGln‐positive cells ± SD.KPolyGln(Arg) proteins but not pure polyGln proteins colocalize with nucleophosmin in HEK293T cells; scale bars: 20 µm; dashed white box in merge panels indicates the region shown as an orthogonal projection in the Ortho zoom panels which show the co‐localization of polyGln inclusions and nucleophosmin in three dimensions. Source data are available online for this figure.

Next, we tested the hypothesis that CGG interruptions increase the toxicity of expanded alleles by affecting RAN and polyglutamine proteins expressed from the CAG repeat. First, we examined whether the arginine interruptions in the polyGln(Arg) proteins increase their toxicity compared with pure polyGln proteins. To perform these experiments, we generated minigene constructs to express polyGln and polyGln(Arg) using non‐hairpin forming (RAN negative) alternative CAA or CAA/AGA‐interrupted codons (Fig 3A). This allowed us to test the toxicity of pure polyGln and interrupted polyGln(Arg) proteins individually and independent of possible effects from CAG expansion RNAs or RAN proteins. We focused these experiments on pure and interrupted polyGln proteins because non‐hairpin forming alternative codons are available for both Gln and Arg. Transient transfections in T98 cells show that interrupted polyGln(Arg) proteins expressed with alternative codons increased cell death by 25% (*P* < 0.05; Fig 3B) and decreased cell viability by 10% compared with pure polyGln proteins (*P* < 0.05; Fig 3C), independent of RNA levels (Fig 3D).

**Figure EV2:**
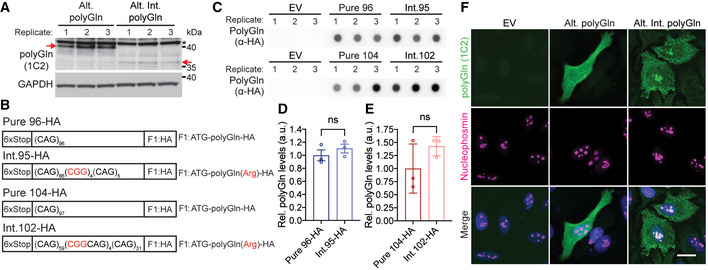
Arginine interruptions alter physical properties of *ATXN8* polyGln protein AProtein blots of transfected HEK293T lysates show polyGln proteins expressed from constructs with interrupted or pure CAA repeat tracts; red arrows indicate pure polyGln and polyGln(Arg) proteins. * The low levels of recombinant protein expressed for toxicity studies allow for polyGln containing TATA‐binding protein to be detected by 1C2 antibody giving a background band at ~ 40 kDa.BConstructs used to express pure and interrupted HA‐tagged polyGln proteins.C–EProtein blot (C) and quantification (D, E) of insoluble protein fraction to detect polyGln proteins by HA epitope tag; EV: empty vector *n* = 3 transfections, ns: not significant, unpaired *t*‐test, mean ± SEM.FPolyGln(Arg) proteins but not pure polyGln proteins colocalize with nucleophosmin in HeLa cells; scale bars: 20 µm. Source data are available online for this figure.

Protein lysates from HEK293T cells overexpressing polyGln(Arg)‐interrupted proteins from alternative codon constructs show polyGln(Arg) proteins migrate further into protein gels than the pure polyGln proteins (Fig EV2A). The change in mobility is likely caused by the introduction of positively charged arginine interruptions in these polar uncharged polyglutamine proteins. Similar results were found in HEK293T cells transfected with CAG repeat constructs (Fig 3E), with quantification showing substantially less soluble interrupted polyGln(Arg) compared with soluble pure polyGln (Fig 3F and G). Dot blots of insoluble protein fractions from HEK293T cells transfected with CAG repeat constructs do not show significant differences in pure polyGln versus polyGln(Arg) levels (Fig EV2B–E).

To further examine the effects of the arginine interruptions, HEK293T cells were transfected with pure and interrupted CAG repeat containing constructs and examined by immunofluorescence (IF). PolyGln(Arg)‐interrupted proteins showed droplet‐like nuclear staining not found in cells expressing pure polyGln proteins (Fig 3H). Similarly, in HEK293T cells transfected with CAA‐ or CAA/AGA‐interrupted constructs, 73% of cells overexpressing polyGln(Arg) proteins have one or more intranuclear droplet‐like inclusions (*P* < 0.0001; Fig 3I and J), which are only rarely (>1%) found in cells overexpressing pure polyGln proteins (Fig 3J). These inclusions, which are found in transfected HEK293T and HeLa cells, colocalize with the nucleolar marker nucleophosmin (Figs 3K and EV2F).

Taken together, these data demonstrate that arginine interruptions promote the formation of droplet‐like nucleolar inclusions and increase the toxicity of polyGln expansion proteins independent of possible CAG RNA gain‐of‐function or RAN protein effects.

### CGG interruptions increase polyAla and polySer RAN protein levels

**Figure 4:**
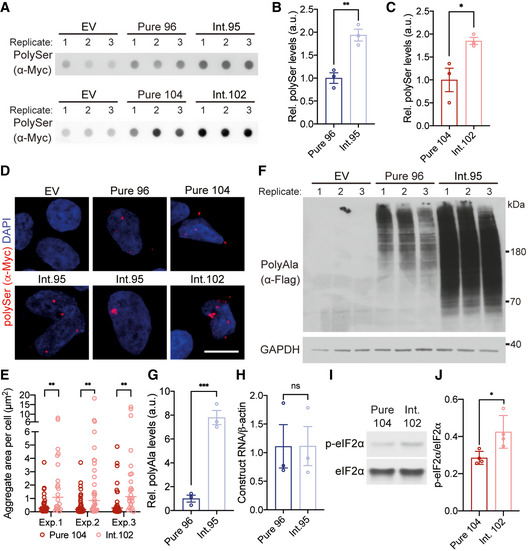
CGG interruptions increase RAN polySer and RAN polyAla protein steady‐state levels A–CProtein blotting (A) and quantification (B, C) of polySer RAN proteins in HEK293T cells from interrupted (Int.95; Int.102) or pure (Pure 96; Pure 104) CAG repeats. EV: empty vector, *n* = 3 transfections, **P < 0.01, *P < 0.05, unpaired *t*‐test, mean ± SEM.D, EImmunofluorescence (D) and quantification (E) of RAN polySer protein aggregates from CGG‐interrupted and pure CAG repeat tracts in HEK293T cells; scale bar: 10 μm. *n* > 20 cells per construct for each experiment (Exp.), **P < 0.01: Exp.1 *P* = 0.0073, Exp.2 *P* = 0.0017, Exp.3 *P* = 0.0041, Mann–Whitney test; data presented as individual points with median.F, GProtein blot (F) and quantification (G) of polyAla RAN proteins expressed from pure or interrupted constructs; *n* = 3 transfections, ***P < 0.001, unpaired *t*‐test, mean ± SD.HRT–qPCR of Pure 96 and Int.95 transcript levels; *n* = 3 transfections; *P* = 0.9942, unpaired *t*‐test, mean ± SEM.I, JProtein blotting (I) and quantification (J) of phospho‐eIF2α (p‐eIF2α) relative to total eIF2α; *n* = 4 transfections, *P < 0.05, unpaired *t*‐test, mean ± SD. Source data are available online for this figure.

**Figure EV3:**
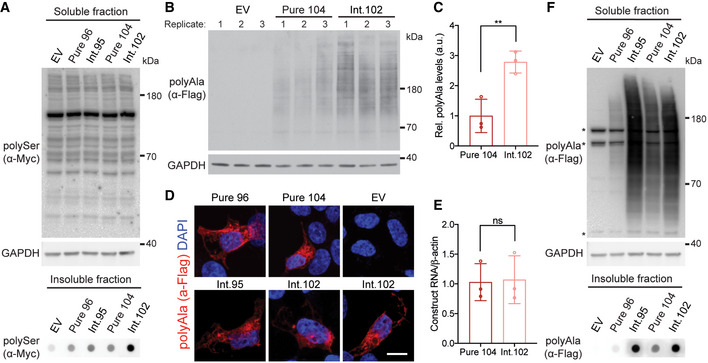
CGG interruptions increase levels of RAN polyAla expansion proteins AProtein blot of soluble and dot blot of insoluble protein fractions for RAN polySer demonstrating that polySer RAN proteins are detectable in the insoluble fraction by dot blot but not the soluble fraction, as no signal above background is found in the soluble fractions; EV: empty vector.B, CProtein blot (B) and quantification (C) of polyAla RAN proteins in HEK293T cells expressed from interrupted (Int.102) and pure (Pure 104) CAG repeat tracts; EV: empty vector; *n* = 3 transfections, **P < 0.01, unpaired *t*‐test, mean ± SD.DImmunofluorescence of RAN polyAla proteins in HEK293T cells; scale bar 10 μm.ERT–qPCR of transcripts expressed from Pure 104 and Int.102 constructs; *n* = 3 transfections; ns: not significant, unpaired *t*‐test, mean ± SEM.FProtein blots of soluble and dot blots of insoluble RAN polyAla proteins expressed from interrupted alleles show increased levels of both soluble and insoluble RAN polyAla compared with those expressed from uninterrupted repeats; * indicates background bands; EV: empty vector. Source data are available online for this figure.

Next, we examined the effects of the CGG interruptions on the two other repetitive proteins encoded by the CAG expansion transcripts, polySer and polyAla RAN proteins. Transient transfections with interrupted and pure repeat constructs show CGG interruptions substantially increase steady‐state levels of polySer and polyAla RAN proteins (Fig 4). In the polySer reading frame, the GGC interruptions produce a polySer protein with glycine interruptions, polySer(Gly). Both pure polySer and polySer(Gly) proteins are highly insoluble with no protein detected in the soluble fraction (Fig EV3A). Dot blot analyses of the insoluble protein fraction showed 93.8% higher levels of interrupted RAN polySer(Gly) compared with pure RAN polySer proteins (*P* < 0.01; Fig 4A and B). Transfections with constructs containing interspersed CGG interruptions (Int.102) showed 85.1% higher levels of RAN polySer(Gly) compared with pure RAN polySer proteins (Pure 104; *P* < 0.05; Fig 4A and C). Similarly, immunofluorescence showed RAN polySer(Gly) proteins form globular or clustered aggregates compared with punctate aggregates formed by pure polySer RAN proteins (Fig 4D). Additionally, total aggregate burden is greater in cells expressing polySer(Arg) compared with pure polySer (*P* < 0.01 for each experiment; Fig 4E).

Protein blots showed even higher increases (7.8‐fold) in steady‐state levels of polyAla RAN proteins expressed from interrupted (Int. 95) compared with pure (Pure 96) CAG repeats (*P* < 0.001; Fig 4F and G). Transfections with constructs containing interspersed CGG interruptions (Int. 102) showed similar polyAla increases (2.8‐fold) compared with size comparable pure repeats (Pure 104; *P* < 0.01; Fig EV3B and C). The increases in polyAla protein levels did not result in overt changes in cellular localization (Fig EV3D) and were found in both soluble and insoluble protein fractions (Fig EV3F). The increases in polyAla and polySer RAN protein levels expressed from interrupted repeats were not caused by differences in RNA levels (Figs 4H and EV3E).

Additionally, we show in transiently transfected HEK293T cells that overexpression of interrupted repeats activated the integrated stress response (ISR) and increased p‐eIF2α levels by 49% compared with pure repeats (*P* < 0.05, Fig 4I and J).

Taken together, these data show CGG interruptions increase steady‐state levels of polySer and polyAla RAN proteins and activate the ISR independent of RNA levels. Additionally, the fact that pure polyAla proteins are expressed from both interrupted and pure CAG expansions indicates that the increase in steady‐state levels of polyAla RAN proteins is not caused by changes in protein composition that could affect the stability of the polyAla protein.

### CGG interruptions increase stability of CAG expansion transcript secondary structure

**Figure 5:**
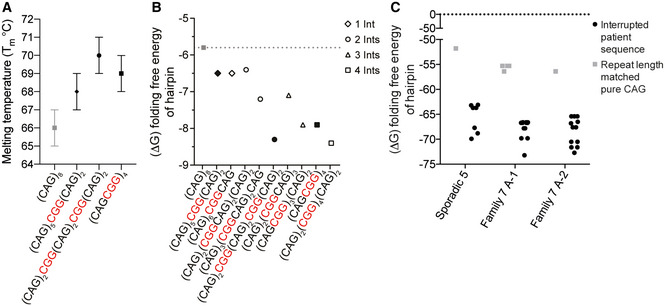
CGG interruptions increase stability of CAG repeat RNA hairpins Absorbance of each RNA substrate at 260 nm monitored between 25 and 95°C, recorded at 1°C intervals; *n* = 3 UV melting curves per RNA substrate (technical replicates), mean ± SD.The m‐fold (Zuker, 2003) predicted folding free energy (Δ*G*) of hairpin structures of pure CAG and CGG‐interrupted repeat tracts with different configurations. Filled symbols show sequences also used for UV melting analyses. Gray dotted line indicates Δ*G* of pure (CAG)_8_.The folding free energy (Δ*G*) of interrupted hairpin structures of repeat expansions found in SCA8 patients (Fig 1I) and pure repeat tracts of the same length, as predicted by m‐fold. Patient‐derived alleles are as follows: Sporadic 5–48 repeats in length—(CAG)_7_(CGGCAG)_18_(CAG)_5_; Family 7 A‐1–53 repeats in length—(CAG)_8_(CGGCAG)_14_(CAG)_2_CGG(CAG)_5_CGG(CAG)_8_; and Family 7 A‐2–52 repeats in length—(CAG)_7_(CGGCAG)_16_(CAG)_4_CGG(CAG)_8_. Each symbol represents a single predicted hairpin structure; multiple hairpin structures, including branched hairpins, are predicted for each SCA8 patient allele and for pure (CAG)_53_ (Zuker, 2003). Black dotted line shows Δ*G* = 0.

**Figure EV4:**
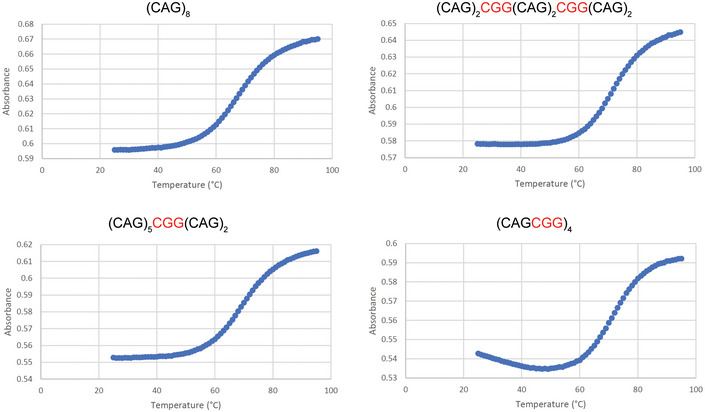
CGG interruptions increase stability of CAG repeat RNA hairpins Example UV melting absorbance curves (for Fig 5A) for pure and interrupted RNA oligos measured at 260 nm monitored between 25 and 95°C, recorded at 1°C intervals.

**Figure EV5:**
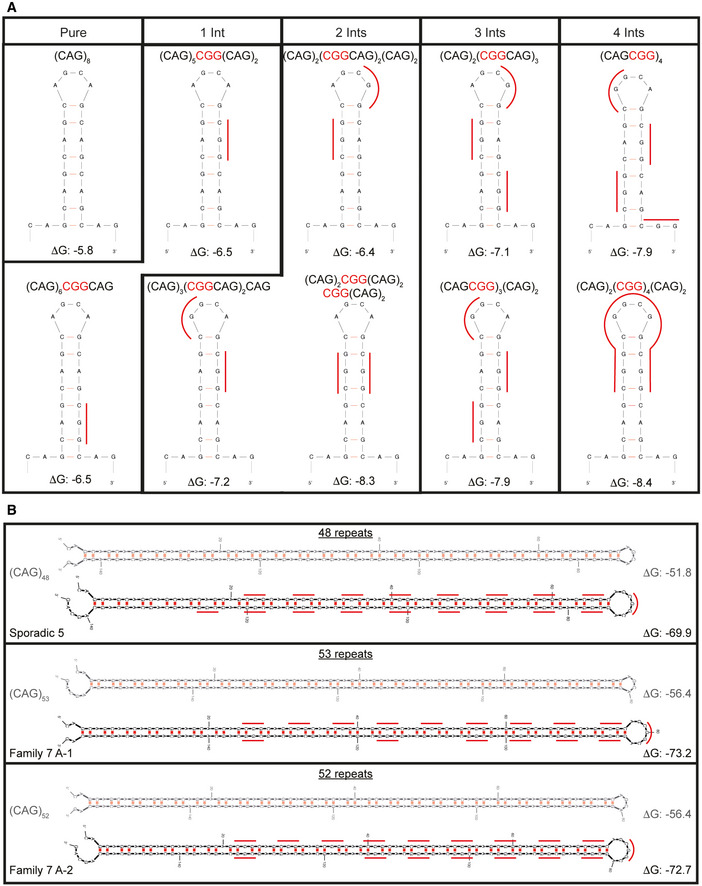
Predicted RNA structures for pure CAG repeat tracts and CGG‐interrupted CAG repeat tracts A, BPredicted RNA hairpin structures from m‐fold (Zuker, 2003) for pure and CGG‐interrupted CAG repeat tracts for Fig 5B (A) and Fig 5C (B). Red lines alongside the structures indicate positions of CGG interruptions. (B) Pure structures are shown in gray; for repeat tracts with multiple predicted hairpin structures, only the most stable structure is shown.

RAN translation is favored by repeat length and RNA structure (Zu *et al*, 2011, 2013; Banez‐Coronel *et al*, 2015; Wang *et al*, 2019), and RNA hairpin stability is known to increase with repeat length (Napierala *et al*, 2005; Wang *et al*, 2019). CGG interruptions increase the steady‐state levels of polyAla without altering the amino acid sequence, suggesting that the increased levels of RAN proteins expressed from interrupted alleles are caused by changes in RNA structure or stability. Consistent with this hypothesis, UV melting analyses of RNA oligos with CGG interruptions required higher melting temperatures than oligos with pure repeats (Figs 5A and EV4). Additionally, computational predictions using *m*‐fold (Zuker, 2003) of short RNAs show increased stability with the presence of CGG interruptions (Figs 5B and EV5A). Next, we examined the stability of interrupted alleles found in patients. We used *m*‐fold to compare the stability of several highly interrupted full‐length CAG repeat tracts from patients (48–53 repeats), with length‐matched pure repeats. Results from these studies show that the multiple predicted hairpin structures, including branched structures, are more stable for alleles containing CGG‐interrupted CAG repeats compared with length‐matched pure CAGs (Figs 5C and EV5B). Both the number and configuration of the interruptions influence RNA structural stability in computational (Fig 5B) and UV melting (Fig 5A) analyses. Taken together, these data are consistent with a model in which increased stability of the secondary structures of CGG‐interrupted expansion transcripts increases RAN translation.

## Discussion

The markedly reduced penetrance is one of the most puzzling features of SCA8 (Koob *et al*, 1999; Stevanin *et al*, 2000; Worth *et al*, 2000; Ikeda *et al*, 2004). Here, we show that 82% of SCA8 families in a large cohort have only a single affected individual, even though the repeat expansion mutation is inherited in an autosomal‐dominant manner. A much smaller percentage of families (13%) showed the expected autosomal‐dominant pattern of disease. CCG•CGG interruptions in the CTG•CAG repeat tract are found at a higher frequency in families with multiple affected individuals and that the number of CCG•CGG interruptions, and not repeat length, correlates with age at onset. Cell culture studies show CAG expansions with CGG interruptions are more toxic than pure repeats. At the protein level, CGG interruptions within the CAG repeat tract increase steady‐state levels of the SCA8 RAN polyAla and polySer proteins. This observation is consistent with the increased stability of RNA structures predicted on CGG‐interrupted alleles and the elevated p‐eIF2α levels. It will be interesting in future work to test whether the elevated p‐eIF2α levels are caused by PKR activation, which can be activated by structured microsatellite RNAs (Edery *et al*, 1989; Tian *et al*, 2000; Zu *et al*, 2020) and which has been recently shown to be a major driver of RAN translation (Zu *et al*, 2020) or result from feedforward effects in which the RAN proteins activate the integrated stress response and p‐eIF2α, which further increases RAN translation (Green *et al*, 2017; Cheng *et al*, 2018; Sonobe *et al*, 2018; Zu *et al*, 2020; Tusi *et al*, 2021). Additionally, CGG interruptions introduce arginine amino acids into the polyGln proteins which increases their toxicity. Taken together, these data demonstrate that CCG•CGG interruptions act as *cis*‐modifiers of SCA8 and provide a molecular explanation for the dramatic variations in disease penetrance among SCA8 families.

We found CCG•CGG interruptions on expanded alleles in all families in our cohort with three or more cases of SCA8. CCG•CGG interruptions were also identified in sporadic SCA8 cases, but at a lower frequency. Additionally, we confirm that repeat length in SCA8 is a poor predictor of disease penetrance (Stevanin *et al*, 2000; Worth *et al*, 2000; Ikeda *et al*, 2004). Taken together, these data indicate that the inclusion of sequence information during genetic testing, specifically the presence or absence of CCG•CGG interruptions, will provide patients and families with additional information relevant to disease penetrance. For asymptomatic SCA8 expansion carriers, the risk of developing ataxia is increased by the presence of CCG•CGG interruptions, which more frequently occur in families with a prior history of ataxia. However, SCA8 ataxia patients may or may not have a family history of disease or sequence interruptions (Table 3). Sequence analyses will also further our understanding of the role of additional types of interruptions on disease penetrance in SCA8 and help identify the causes of high penetrance in other large SCA8 families in the literature for which the expansion sequences are unknown (Cintra *et al*, 2017). Additionally, we have identified SCA8 patients with shorter repeat expansions than have been previously reported, expanding the range of repeats found in individuals affected with ataxia to 54–1,455 repeats.

**Table 3:** Lifetime risk of developing SCA8.

*ATXN8OS*/*ATXN8* genotype		Ataxia symptoms	Lifetime risk of developing SCA8
CTG•CAG expansion	+ CCG•CGG interruptions		
+	−	−	At risk
+	+	−	Increased risk
+	+/‐	+	SCA8

A CTG•CAG expansion in *ATXN8OS/ATXN8* is sufficient to put an individual at risk for developing SCA8. For asymptomatic SCA8 expansion carriers, the risk of developing ataxia is increased by the presence of CCG•CGG interruptions, which more frequently occur in families with a prior history of ataxia The presence or absence of interruptions would not influence a diagnosis of SCA8 in patients with ataxia symptoms and SCA8 CTG•CAG expansions, including diagnosis of sporadic SCA8 cases.

Here, we show that polyGln(Arg)‐interrupted proteins are more toxic than pure polyGln proteins and that steady‐state levels of RAN polyAla and polySer proteins expressed from interrupted repeats are increased. This increase in toxicity caused by CCG•CGG interruptions could be sufficient to tip the balance of cellular homeostasis to degeneration and cell death in cells sensitive to the SCA8 repeat expansion. Taken together, it is possible that CCG•CGG interruptions in SCA8 patients increase overall cellular toxicity and RAN protein load which may in turn exacerbate the associated pathologies, including white matter defects (Ayhan *et al*, 2018), in SCA8. While the data presented here provide insight into possible molecular consequences of the CCG•CGG interruptions in SCA8 repeat expansions, further detailed analyses in patient cell lines and postmortem tissue will be necessary to fully understand the pathological consequences of the CCG•CGG interruptions. Additionally, while the current SCA8‐BAC mouse model carries CCG•CGG interruptions, development of an isogenic SCA8 expansion mouse model without CCG•CGG interruptions would be valuable to further investigate the role of CCG•CGG interruptions on disease in SCA8. Directly comparing tissues and cell lines from SCA8 patients or animal models with pure and CCG•CGG‐interrupted repeat expansions would also improve our understanding of the contribution of repeat expansion proteins to disease.

RAN translation has now been described in 11 microsatellite expansion diseases and in several of these diseases the differences between RAN proteins that contain arginine residues and those that do not have been characterized. For example, in myotonic dystrophy type 2 (DM2), RAN translation across the sense CCTG repeat produces polyLPAC proteins which show a diffuse cytoplasmic distribution in HEK293T cells but RAN translation across the antisense CAGG repeat produces polyQAGR proteins which form droplet‐like nuclear staining not found with polyLPAC proteins (Zu *et al*, 2017). Similarly, in *C9orf72* ALS/FTD, polyGR and polyPR form droplet‐like nuclear structures that colocalize with nucleolar markers while polyGA, polyGP, and polyPA show diffuse nuclear and cytoplasmic localization (Wen *et al*, 2014; Tao *et al*, 2015). Furthermore, the arginine containing polyGR and polyPR proteins are more toxic in cell culture and Drosophila than the dipeptide repeat proteins that do not contain arginine residues (Mizielinska *et al*, 2014; Wen *et al*, 2014; Tao *et al*, 2015; Lee *et al*, 2016) and they have been shown to impair ribosomal RNA biogenesis, perturb stress granule dynamics and biomolecular phase separation, and induce nucleolar stress (Kwon *et al*, 2014; Tao *et al*, 2015; Lee *et al*, 2016; Boeynaems *et al*, 2017; White *et al*, 2019). Similar to the polyGR and polyPR proteins found in *C9orf72* ALS/FTD, the SCA8 arginine‐interrupted polyGln(Arg) proteins show increased toxicity and droplet‐like nucleolar inclusions. These striking molecular parallels between SCA8, DM2, and *C9orf72* ALS/FTD suggest similar molecular mechanisms are at play and that the effects of polyGln(Arg) proteins on nucleolar stress and stress granule dynamics warrant further investigation.

There is a growing body of evidence that structured RNAs, including RNA hairpins, favor efficient RAN translation (Zu *et al*, 2011; Banez‐Coronel *et al*, 2015; Wang *et al*, 2019). RAN translation has also been shown to be more efficient with longer repeat lengths, and longer repeats increase the stability of RNA secondary structures (Napierala *et al*, 2005; Zu *et al*, 2011, 2013; Banez‐Coronel *et al*, 2015; Wang *et al*, 2019; Pattamatta *et al*, 2020). Our data extend these results and show that CGG interruptions, which increase the stability of RNA hairpins, also lead to elevated levels of RAN proteins and show that increasing RNA stability without altering repeat tract length increases RAN translation. Additionally, the increased stability of RNA secondary structures containing CGG interruptions could also lead to increased toxicity through RNA gain‐of‐function mechanisms (Daughters *et al*, 2009) possibly by the changes in the sequestration of known or novel RNA binding proteins by SCA8 expansion transcripts.

While additional types of AT‐rich sequence interruptions (e.g., CTT•AAG, CCA•TGG, CTA•TAG) have been reported in SCA8 (Moseley *et al*, 2000b; Hu *et al*, 2017), the lack of highly penetrant SCA8 families with AT‐rich interruptions (Moseley *et al*, 2000b) makes it unlikely that they increase disease penetrance in a manner similar to CGG interruptions. This is consistent with the prediction that AT‐rich interruptions decrease RNA structural stability of CAG expansion transcripts in contrast to CGGs, which increase RNA secondary structure stability. A small number of sporadic cases are homozygous for the expansions, suggesting the presence of two SCA8 expansion alleles may also increase disease penetrance (Fig 1B). The fact that SCA8 is also found with reduced penetrance in patients with single uninterrupted expansion mutations suggests that, similar to other neurodegenerative diseases, *trans*‐genetic modifiers and environmental factors are also likely to contribute to disease (Mo *et al*, 2015; Hosseinibarkooie *et al*, 2017).

In summary, CCG•CGG interruptions within the SCA8 CAG repeat tract are associated with increased penetrance in SCA8 families. At the molecular level, CCG•CGG interruptions increase RNA stability and levels of polyAla and polySer RAN proteins. Additionally, CCG•CGG interruptions encode alternative amino acids that increase the toxicity and change the molecular properties of the resulting polyGln(Arg) proteins. Taken together, these data provide novel insight into the molecular mechanisms affecting disease penetrance in SCA8.

## Materials and Methods

### Research participants

Informed consent was acquired from all participants in accordance with the Human Subjects Committee at the University of Minnesota, the Institutional Review Board (IRB) at the University of Florida, or the equivalent office at collaborators' institutions. The experiments conformed to the principles set out in the WMA Declaration of Helsinki and the Department of Health and Human Services Belmont Report. A board‐certified neurologist identified SCA8 probands on clinical examination, and interested patients were enrolled into the research study. Family history of ataxia was assessed by questionnaire, and patients were encouraged to inform affected and unaffected relatives of the research study; volunteers were enrolled into the study. SCA8 families were included in our study if the repeat length and gender of the proband were known. Patients for whom repeat size could not be determined or DNA was not available were not included in the study. Samples were collected from 77 independent families.

### Genetic analysis of SCA8 repeat expansions

Genomic DNA (gDNA) was extracted from peripheral blood lymphocytes using FlexiGene DNA Kit (Qiagen). The number of combined CTG•CAG/CTA•TAG repeats at the SCA8 locus was determined by PCR across the repeat using CAG‐1F (5′ TTT GAG AAA GGC TTG TGA GGA 3′) and CAG‐1R (5′ TCT GTT GGC TGA AGC CCT AT 3′) primers. PCR bands were extracted using Wizard SV Gel and PCR Clean‐Up System (Promega) and, when possible, sent for direct DNA sequencing using nested primers CAG‐3F (5′ GGC TTG TGA GGA CTG AGA ATG 3′) and CAG‐3R (5′ GAA GCC CTA TTC CCA ATT CC 3′). Expansions too large for direct sequence (approximately > 250 repeats) were digested with MspA1I (New England Biolabs) which ambiguously digests the PCR products containing either CGG or CTG interruptions in the CAG direction of the repeat tract. This method confirms the presence of CGG interruptions but does not provide the sequence configuration. If the expansion size was too large to perform PCR across the repeat, the repeat length was estimated by Southern blot. Families found to have non‐CGG interruptions were excluded from analysis of CGG interruptions and disease penetrance.

### cDNA constructs

To generate patient‐derived pure and interrupted SCA8 expansion constructs (Pure 104, Int.102, Pure 96, Int.95) for molecular characterization of CGG interruptions, a region containing the *ATXN8* open reading frame was PCR amplified from patients' gDNA using primers SCA8‐F3‐Kpn1 (5′ TTG GTA CCT TTG AGA AAG GCT TGT GAG GAC TGA GAA TG 3′) and SCA8‐R4‐EcoRI (5′ GCG AAT TCG GTC CTT CAT GTT AGA AAA CCT GGC T 3′). The PCR fragment was cloned in the CAG direction into the pcDNA3.1‐6xStop‐triple tag (pcDNA3.1‐6S‐3T) vector which has a six stop cassette (two stop codons per reading frame) upstream of the repeat and a unique C‐terminal tag in each reading frame (Zu *et al*, 2011). The pcDNA3.1‐6S‐3T construct was used as the empty vector for all studies. Due to the TAG repeat tract encoding for multiple stop codons after the CAG repeat stretch, there is no C‐terminal tag in the CAG frame. Construct names (Pure 104, Int.102, Pure 96, Int.95) denote the total CAG tract length which, due to repeat instability during cloning, may not be the same total tract length as the patient alleles used to clone the repeat sequences.

To assess polyGln proteins in the insoluble fraction, the CAG expansion constructs were re‐cloned without the TAG repeat tract (GenScript) to generate HA‐tagged polyGln proteins which can be detected by a HA‐tag antibody. While the repeat configuration is identical between the constructs with and without the TAG codons, due to repeat instability during cloning, the repeat tract length may not be identical (Fig EV2B).

To assess toxicity of polyGln proteins, ATG‐initiated non‐hairpin forming alternative codon minigenes were synthesized by IDT Technologies and subcloned into the pcDNA3.1‐6S‐3T vector. PolyGln is encoded by CAA repeats with AGA‐encoded Arginine interruptions to generate the Alt. polyGln and Alt. Int. polyGln constructs (Fig 3A). It is not possible to model polyAla or polySer proteins using this system as no non‐hairpin forming alternative codons exist for alanine or glycine.

### Cell culture and transfections

HEK293T, T98, or HeLa cells were cultured in DMEM (Corning) supplemented with 10% fetal bovine serum (Gibco) and 1X penicillin–streptomycin (Gibco). Plasmid transfections were performed using Lipofectamine 2000 (Invitrogen) according to the manufacturer's instructions. Plasmid transfection amounts were optimized for each set of constructs used for toxicity assays. For polyGln IF analyses, 4 × 10^4^ cells were seeded per well of a poly‐d‐lysine/laminin coated eight‐well culture slide (Corning) and transfected with 150 ng construct DNA using 0.25 μl Lipofectamine 2000 per well.

### Toxicity and viability assays

Cell toxicity and viability were assessed in T98 cells 42 h post‐transfection using the CytoTox 96 Nonradioactive Cytotoxicity Assay (Promega) or 3‐(4,5‐dimethyl‐thiazol‐2‐yl)‐2,5‐diphenyl tetrazolium bromide (MTT) assay (Sigma), respectively, following the manufacturer's protocol. Briefly, total LDH release was measured by lysing the cells with 1% Triton X‐100 and absorbance was measured at 490 nm. MTT was added to cell culture media at a final concentration of 0.5 mg/ml and incubated for 45 min at 37°C. Following media removal cells were lysed with 100 μl of dimethyl sulfoxide (DMSO; Fisher Scientific), and absorbance was measured at 595 nm. Toxicity and viability assays were performed in a minimum of six independent experiments, and in each independent experiment, the assays were performed in quintuplet.

### RNA extraction and RT–qPCR

RNA was isolated from transiently transfected HEK293T or T98 cells using TRIzol Reagent (Invitrogen). RNA was DNase treated using TURBO DNA‐free Kit (Ambion), following the manufacturer's instructions. cDNA was synthesized using random hexamer primers and the SuperScript III Reverse Transcriptase System (Invitrogen) following the manufacturer's protocol. Quantification of construct transcript levels was performed using the 5'FLAG (5′ GAT TAC AAG GAC GAC GAC GAC 3′) and 3'HIS (5′ ATG GTG ATG GTG ATG ATG ACC 3′) primers. Control reactions were performed using human β‐actin forward (5′ TCG TGC GTG ACA TTA AGG AG 3′) and human β‐actin reverse (5′ GAT CTT CAT TGT GCT GGG TG 3′) primers. For each biological replicate, three technical replicates were performed for qPCR. RT–qPCR results were analyzed using the $2^{‐\Delta \Delta \text{CT}}$ method (Livak & Schmittgen, 2001).

### Immunoblotting

HEK293T cells were washed with 1xPBS 48 h post‐transfection and were lysed in 200 μl radioimmunoprecipitation assay (RIPA) buffer (Thermo Scientific) with 1X cOmplete Protease Inhibitors (Roche) for 15 min on ice. DNA was sheared by passage through a 21‐gauge needle, lysates were centrifuged at 21,000 *g* for 15 min at 4°C, and the supernatant was collected. The protein lysate concentration was quantified using Pierce BCA Protein Assay Kit (Thermo Scientific), and 10μg of soluble protein lysates was separated on a 4–12% Bis–Tris gel (Bio‐Rad) and transferred to a nitrocellulose membrane. The remaining insoluble protein pellet was extracted in 2% SDS in RIPA buffer by incubating at 42°C for 3 h with frequent repeated pipetting, incubated at room temperature overnight, centrifuged at 21,000 *g* for 15 min at room temperature, and the supernatant was collected. Insoluble protein lysate was passed through a Dot Blot Apparatus (Bio‐Rad) onto a PVDF membrane. Membranes were blocked for 2 h at room temperature in 5% dry milk in 1xPBS containing 0.05% Tween‐20 (Sigma) and probed with anti‐FLAG antibody (Abcam, Cat. # ab1162, 1:2,000), anti‐myc antibody (Abcam, Cat. # ab9106, 1:1,000), anti‐HA antibody (BioLegend, Cat. # 901501, clone 16B12, 1:2,000), 1C2 antibody (Millipore, Cat. # MAB1574, 1:10,000), rabbit anti‐EIF2S1 (phospho‐S51; Abcam, Cat. # ab32157, 1:1,000), mouse anti‐eIF2α (Santa Cruz, sc‐133227, 1:2,000), and anti‐GAPDH antibody (Millipore, Cat. # MAB374, 1:5,000) overnight at 4°C in blocking solution. The membrane was incubated with species‐specific HRP‐conjugated secondary antibody (Amersham) in blocking solution, and bands were visualized with the ECL plus Western Blotting Detection System (Amersham). Quantification of protein expression was performed using ImageJ. For dot blot quantification antibody signal for empty vector transfections was used to perform background reduction. All protein levels are normalized to pure repeat expansion protein levels.

### Immunofluorescence

HEK293T and HeLa cells were fixed 48 h post‐transfection (for RAN polyAla and RAN polySer) or 24 h post‐transfection (for polyGln) with 4% paraformaldehyde (PFA; Electron Microscopy Sciences) in 1 × PBS for 15 min and permeabilized with 0.5% Triton X‐100 (Sigma) in 1 × PBS for 30 min. Cells were blocked in 1% normal goat serum (NGS) for 30 min and incubated overnight at 4°C with 1C2 antibody (Millipore, Cat. # MAB1574, 1:10,000), nucleophosmin antibody (Abcam, Cat. # ab183340, 1:500), alpha‐tubulin (Abcam, Cat. # ab52866, 1:500), or anti‐FLAG antibody (Abcam, Cat. # ab1162, 1:1,000), or for 1 h at 37°C with anti‐myc antibody (Abcam, Cat. # ab9106, 1:1,000). Cells were incubated with AlexaFluor‐conjugated secondary antibodies (1:300) for 1 h at room temperature and were mounted with ProLong Gold Antifade (Thermo Scientific). Representative images were taken using the LSM 880 (ZEISS) confocal microscope with an AiryScan module. Images for quantification were taken using the LSM 880 (ZEISS) confocal microscope using consistent settings between experiments. For analysis of polyGln intranuclear inclusions, 1C2 staining was used to determine whether cells were positive for polyGln proteins, and then, the number of intranuclear inclusions was quantified. Analysis of polySer RNA protein aggregates was performed in ImageJ using the MaxEntropy threshold followed by the Smooth, Make Binary, and Analyze Particles functions.

### UV melting

RNA oligonucleotides were purchased from IDT. Absorbance of each RNA substrate at 260nm was monitored between 25 and 95°C, recorded at 1°C intervals. Three UV melting curves were generated per RNA substrate at a concentration of 2 µM in 1xDPBS without calcium or magnesium.

### Statistical analysis

All statistical analyses were performed using GraphPad Prism 6.0 software. The D'Agostino and Pearson normality test or Shapiro–Wilk normality test were used to assess normal distribution and statistical tests for which the data meet the assumptions of the tests were selected. Statistical relationship of CCG•CGG interruptions and disease penetrance was calculated using Fisher's exact test. Linear regression analyses were performed to assess the relationship between age of onset and repeat length or interruption number. All other statistical analyses were performed using an unpaired two‐tailed *t*‐test or a two‐way ANOVA for parametric data or the Mann–Whitney test for non‐parametric data, as appropriate. Investigators were blinded for all quantification of immunofluorescent data. All cell culture experiments were performed with a minimum of 3 biological replicates; the variation in cell‐based systems was consistent across multiple transfections. Randomization was not applicable to this study as samples were not assigned to groups. Data are reported as mean ± SEM, mean ± SD, individual values, or median.

## Author contributions

BAP, HKS, YI, and LPWR designed the project. HKS, BAP, MB‐C, LELR, TZ, TR, LAL, KR, and YI conducted experiments. HKS, BAP, YI, MB‐C, KR, and JAB analyzed data. SHS, CMG, TA, TB, AB, LJS, LFH, JEN, and LPWR provided patient DNA samples and collected clinical information. HKS, BAP, and LPWR wrote the manuscript with input from all the authors.

## Conflict of interests

Tao Zu and Laura Ranum are inventors on patents and pending patents on RAN translation and/or SCA8.

## For more information

https://neurogenetics.med.ufl.edu/faculty/dr‐laura‐p‐w‐ranum/

https://www.ataxia.org/fact‐sheets/

https://www.omim.org/entry/608768

https://www.ncbi.nlm.nih.gov/books/NBK1268/

## Supporting information



Appendix

Expanded View Figures PDF

Source Data for Expanded View

Source Data for Figure 3

Source Data for Figure 4

## Data Availability

This study includes no data deposited in public repositories. All data is available in the source files or appendix.
