# Supplementary material for: CCG•CGG interruptions in high‐penetrance SCA8 families increase RAN translation and protein toxicity
Source: EMBO Mol Med. 2021 Oct 11;13(11):e14095. doi: 10.15252/emmm.202114095 (PMC8573593; doi:10.15252/emmm.202114095)
Supplement: Supplementary file 1 — Appendix [file EMMM-13-e14095-s003.pdf]

CCG•CGG interruptions in high penetrance SCA8 families increase RAN translation and protein toxicity

Appendix

Table of Contents

Appendix Table S1: Repeat configurations of CCG•CGG interrupted *ATXN8OS/ATXN8* CTG•CAG expanded alleles in order of Figure 1I from Sporadic 1A to Family 7 AS 2

Appendix Table S2: Configurations for pure ATXN8OS/ATXN8 CTG•CAG expanded alleles

Appendix Table S3: Configurations for ATXN8OS/ATXN8 CTG•CAG expanded alleles that contain non-CCG•CGG interruptions

Note: Allele configurations are not available for individuals determined to carry pure or CCG•CGG interrupted *ATXN8OS/ATXN8* CTG•CAG expanded alleles by restriction digest, therefore, these individuals are not included in Appendix Tables S1-3. Allele configurations for both expanded alleles from homozygous patients are included where possible and denoted as Allele 1 and Allele 2.

Appendix Table S1: Repeat configurations of CCG•CGG interrupted *ATXN8OS/ATXN8* CTG•CAG expanded alleles in order of Figure 1I from Sporadic 1A to Family 7 AS 2

Individual	Allele Configuration
Sporadic 1A	$(CAG)_{91}(CAGCGG)_{3}(CAG)_{31}(TAG)_{10}$
Sporadic 2A	$(CAG)_{93}CGG(CAG)_{2}CGG(CAG)_{6}CGG(CAGCAGCGG)_{2}(CAG)_{20}(TAG)_{13}$
Sporadic 3A	$(CAG)_{43}CGG(CAG)_{32}(TAG)_{7}$
Sporadic 4A	$(CAG)_{14}CGG(CAG)_{4}(CGGCAG)_{2}(CGG)_{2}(CAGCGG)_{2}(CAG)_{122}(TAG)_{3\text{-}21}$
Sporadic 5A	$(CAG)_{6}(CAGCGG)_{18}(CAG)_{6}(TAG)_{6}$
Family 1A	$(CAG)_{118}CGG(CAG)_{5}(TAG)_{3}$
Family 1A	$(CAG)_{104}CGG(CAG)_{7}CGG(CAG)_{5}(TAG)_{3}$
Family 1A	$(CAG)_{112}CGG(CAG)_{5}(TAG)_{3}$
Family 1A	$(CAG)_{114}CGG(CAG)_{2}CGG(CAG)_{5}(TAG)_{3}$
Family 1A	$(CAG)_{107}CGG(CAG)_{2}CGG(CAG)_{5}(TAG)_{3}$
Family 1A	$(CAG)_{120}CGG(CAG)_{5}(TAG)_{3}$
Family 1A	$(CAG)_{100}CGG(CAG)_{5}(TAG)_{3}$
Family 1A	$(CAG)_{109}CGG(CAG)_{5}(TAG)_{3}$
Family 1A	$(CAG)_{105}(CGG)_{3}(CAG)_{5}(TAG)_{3}$
Family 1A	$(CAG)_{81}(CGG)_{3}(CAG)_{5}(TAG)_{3}$
Family 1A	Allele 1: $(CAG)_{103}(CGG)_{3}(CAG)_{5}(TAG)_{3}$ / Allele 2: $(CAG)_{81}(TAG)_{3}$
Family 1A	$(CAG)_{108}CGG(CAG)_{5}(TAG)_{3}$
Family 1AS	$(CAG)_{83}CGG(CAG)_{2}CGG(CAG)_{5}(TAG)_{3}$
Family 1AS	$(CAG)_{137}CGG(CAG)_{5}(TAG)_{3}$
Family 1AS	$(CAG)_{91}CGG(CAG)_{5}(TAG)_{3}$
Family 1AS	$(CAG)_{80}CGG(CAG)_{5}(TAG)_{3}$
Family 1AS	$(CAG)_{86}(CGG)_{3}(CAG)_{5}(TAG)_{3}$
Family 1AS	$(CAG)_{87}(CGG)_{4}(CAG)_{5}(TAG)_{3}$
Family 1AS	$(CAG)_{85}(CGG)_{2}(CAG)_{5}(TAG)_{3}$
Family 1AS	$(CAG)_{64}CGG(CAG)_{9}CGG(CAG)_{7}(CGG)_{2}(CAG)_{5}(TAG)_{3}$
Family 1AS	$(CAG)_{92}(CGG)_{4}(CAG)_{5}(TAG)_{3}$
Family 2A	$(CAG)_{59}(CAGCGG)_{5}(CAG)_{35}(TAG)_{6}$
Family 2A	$(CAG)_{57}(CAGCGG)_{6}(CAG)_{35}(TAG)_{6}$
Family 2A	$(CAG)_{57}(CAGCGG)_{5}(CAG)_{35}(TAG)_{6}$
Family 2A	$(CAG)_{61}(CAGCGG)_{4}(CAG)_{34}(TAG)_{6}$
Family 2A	$(CAG)_{56}(CAGCGG)_{6}(CAG)_{33}(TAG)_{6}$
Family 2AS	$(CAG)_{53}(CAGCGG)_{7}(CAG)_{2}(CAGCGG)_{4}(CAG)_{25}(TAG)_{6}$
Family 2AS	$(CAG)_{53}(CAGCGG)_{4}(CAG)_{32}(TAG)_{6}$

Family 3A	$(CAG)_{78}((CAG)_{2}CGG)_{9}(CAG)_{114}(TAG)_{8}$
Family 4A	$(CAG)_{80}CGG(CAG)_{9}CGG(CAG)_{6}CGG(CAG)_{19}GAG(TAG)_{8}$
Family 4A	$(CAG)_{80}CGG(CAG)_{9}CGG(CAG)_{6}CGG(CAG)_{19}GAG(TAG)_{8}$
Family 5A	$(CAG)_{63}((CAG)_{3}CGG)_{4}(CAG)_{6}CGG((CAG)_{3}CGG)_{2}(CAG)_{98}(TAG)_{10}$
Family 5A	$(CAG)_{69}((CAG)_{3}CGG)_{4}(CAG)_{2}CGG((CAG)_{3}CGG)_{3}(CAG)_{106}(TAG)_{10}$
Family 6A	$(CAG)_{67}(CGG)_{5}(CAG)_{5}CGG(CAG)_{97}(TAG)_{9}$
Family 6A	$(CAG)_{63}(CGG)_{8}(CAG)_{97}(TAG)_{12}$
Family 7A	$(CAG)_{7}(CAGCGG)_{14}(CAG)_{3}CGG(CAG)_{5}CGG(CAG)_{8}(TAG)_{9}$
Family 7A	$(CAG)_{6}(CAGCGG)_{16}(CAG)_{5}CGG(CAG)_{8}(TAG)_{9}$
Family 7AS	$(CAG)_{7}(CAGCGG)_{19}(CAG)_{8}(TAG)_{9}$
Family 7AS	$(CAG)_{6}(CAGCGG)_{19}(CAG)_{8}(TAG)_{9}$

Appendix Table S2: Configurations for pure *ATXN8OS/ATXN8* CTG•CAG expanded alleles

Affected expansion carriers	Asymptomatic expansion carriers
$(CAG)_{283}(TAG)_{11}$	$(CAG)_{225}(TAG)_{12}$
$(CAG)_{79}(TAG)_{10}$	$(CAG)_{75}(TAG)_{11}$
$(CAG)_{76}(CAG)_{9}$	$(CAG)_{75}(TAG)_{11}$
$(CAG)_{79}(TAG)_{13}$	$(CAG)_{71}(TAG)_{11}$
$(CAG)_{81}(TAG)_{13}$	$(CAG)_{75}(TAG)_{11}$
$(CAG)_{174}(TAG)_{12}$	$(CAG)_{88}(TAG)_{13}$
$(CAG)_{65}(TAG)_{10}$	$(CAG)_{114}(TAG)_{13}$
$(CAG)_{133}(TAG)_{8}$	$(CAG)_{80}(TAG)_{13}$
$(CAG)_{84}(TAG)_{9}$	$(CAG)_{119}(TAG)_{12}$
$(CAG)_{90}(TAG)_{8}$	$(CAG)_{92}(TAG)_{3}$
$(CAG)_{93}(TAG)_{7}$	$(CAG)_{69}(TAG)_{9}$
$(CAG)_{85}(TAG)_{7}$	$(CAG)_{86}(TAG)_{3}$
$(CAG)_{85}(TAG)_{9}$	$(CAG)_{70}(TAG)_{9}$
$(CAG)_{67}(TAG)_{13}$	$(CAG)_{94}(TAG)_{3}$
$(CAG)_{65}(TAG)_{11}$	$(CAG)_{85}(TAG)_{6}$
$(CAG)_{137}(TAG)_{8}$	$(CAG)_{94}(TAG)_{3}$
$(CAG)_{60}(TAG)_{23}$	$(CAG)_{88}(TAG)_{3}$
$(CAG)_{95}(TAG)_{11}$	$(CAG)_{87}(TAG)_{4}$
$(CAG)_{115}(TAG)_{7}$	$(CAG)_{101}(TAG)_{3}$
$(CAG)_{77}(TAG)_{11}$	$(CAG)_{91}(TAG)_{3}$
$(CAG)_{125}(TAG)_{8}$	$(CAG)_{80}(TAG)_{3}$
$(CAG)_{118}(TAG)_{10}$	$(CAG)_{99}(TAG)_{3}$
$(CAG)_{256}(TAG)_{{}^{3}4}$	$(CAG)_{135}(TAG)_{8}$
$(CAG)_{77}(TAG)_{9}$	$(CAG)_{70}(TAG)_{9}$
$(CAG)_{97}(TAG)_{9}$	$(CAG)_{69}(TAG)_{11}$
$(CAG)_{94}(TAG)_{10}$	$(CAG)_{70}(TAG)_{9}$
$(CAG)_{87}(TAG)_{11}$	$(CAG)_{81}(TAG)_{10}$
$(CAG)_{51}(TAG)_{9}$	$(CAG)_{92}(TAG)_{7}$
$(CAG)_{65}(TAG)_{15}$	$(CAG)_{118}(TAG)_{10}$
$(CAG)_{104}(TAG)_{9}$	$(CAG)_{79}(TAG)_{8}$
$(CAG)_{96}(TAG)_{9}$	$(CAG)_{85}(TAG)_{10}$
$(CAG)_{80}(TAG)_{12}$	$(CAG)_{85}(TAG)_{7}$
$(CAG)_{88}(TAG)_{10}$	$(CAG)_{136}(TAG)_{14}$
$(CAG)_{81}(TAG)_{12}$	$(CAG)_{117}(TAG)_{15}$

$(CAG)_{78}(TAG)_{12}$	$(CAG)_{108}(TAG)_{17}$
$(CAG)_{81}(TAG)_{8}$	$(CAG)_{95}(TAG)_{10}$
$(CAG)_{89}(TAG)_{8}$	$(CAG)_{74}(TAG)_{8}$
$(CAG)_{91}(TAG)_{9}$	$(CAG)_{91}(TAG)_{12}$
	$(CAG)_{236}(TAG)_{9}$
	$(CAG)_{84}(TAG)_{12}$
	$(CAG)_{92}(TAG)_{5}$
	$(CAG)_{77}(TAG)_{9}$

Appendix Table S3: Configurations for *ATXN8OS/ATXN8* CTG•CAG expanded alleles that contain non-CCG•CGG interruptions

Affected expansion carriers	Asymptomatic expansion carriers
$(CAG)_{72}TAGTGG(TAG)_{15}$	$(CAG)_{67}TAGTGG(TAG)_{15}$
Allele 1: $(CAG)_{117}TAGCAG(TAG)_{11}$ / Allele 2: $(CAG)_{95}(TAG)_{9}$	$(CAG)_{68}TAGTGG(TAG)_{15}$
$(CAG)_{133}(TAGCAG)_{3}(TAG)_{11}$	$(CAG)_{56}TAG(CAG)_{3}(TAGCAG)_{9}(TAG)_{12}$
$(CAG)_{55}TAG(CAG)_{3}(TAGCAG)_{9}(TAG)_{12}$	$(CAG)_{61}(TAG)_{2}TAC(CAG)_{2}(TAG)_{10}$
$(CAG)_{40}CTG(CAG)_{18}(TAG)_{10}$	$(CAG)_{41}(CTG)_{2}(CAG)_{18}(TAG)_{11}$
$(CAG)_{18}CCG(CAG)_{5}CCG(CAG)_{7}CCG(CAG)_{86}(TAG)_{8}$	$(CAG)_{69}(TAG)_{2}(TAGCAG)_{2}(TAG)_{9}$
$(CAG)_{65}TAG(TAGCAG)_{2}(TAG)_{9}$	$(CAG)_{65}(TAG)_{2}(TAGCAG)_{2}(TAG)_{9}$
$(CAG)_{114}TAGCAG(TAG)_{11}$	$(CAG)_{67}TAG(TAGCAG)_{2}(TAG)_{9}$
$(CAG)_{102}AAG(CAG)_{2}(TAG)_{9}$	$(CAG)_{80}GAG(TAG)_{8}$
One additional allele was found to carry TAG interruptions by sequencing but due to the length of the expansion (447 repeats), the allele configuration could not be determined.	
