# Supplementary material for: CCG•CGG interruptions in high‐penetrance SCA8 families increase RAN translation and protein toxicity
Source: EMBO Mol Med. 2021 Oct 11;13(11):e14095. doi: 10.15252/emmm.202114095 (PMC8573593; doi:10.15252/emmm.202114095)
Supplement: Supplementary file 2 — Expanded View Figures PDF [file EMMM-13-e14095-s002.pdf]

Expanded View Figures

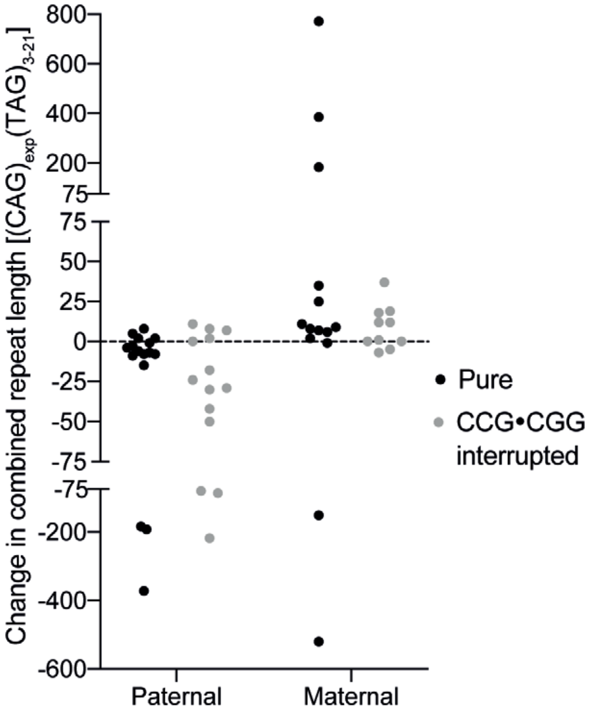

Figure EV1. CCG•CGG interruptions do not affect inheritance patterns of SCA8 CTG•CAG repeat expansions.

No differences in repeat length changes for paternal or maternal transmissions of pure versus CCG•CGG-interrupted alleles. Paternal transmission of pure alleles: $n = 16$, CCG•CGG-interrupted alleles: $n = 14$. Maternal transmission of pure alleles: $n = 14$, CCG•CGG-interrupted alleles: $n = 10$. Individuals carrying two expanded alleles were included if sequencing could determine transmitted allele. No significant differences in repeat length changes were found for pure versus interrupted transmissions of paternal ($P = 0.5314$) or maternal alleles ($P = 0.5748$) using Mann–Whitney test. Dashed line represents no change in repeat length.

 © 2021 The Authors

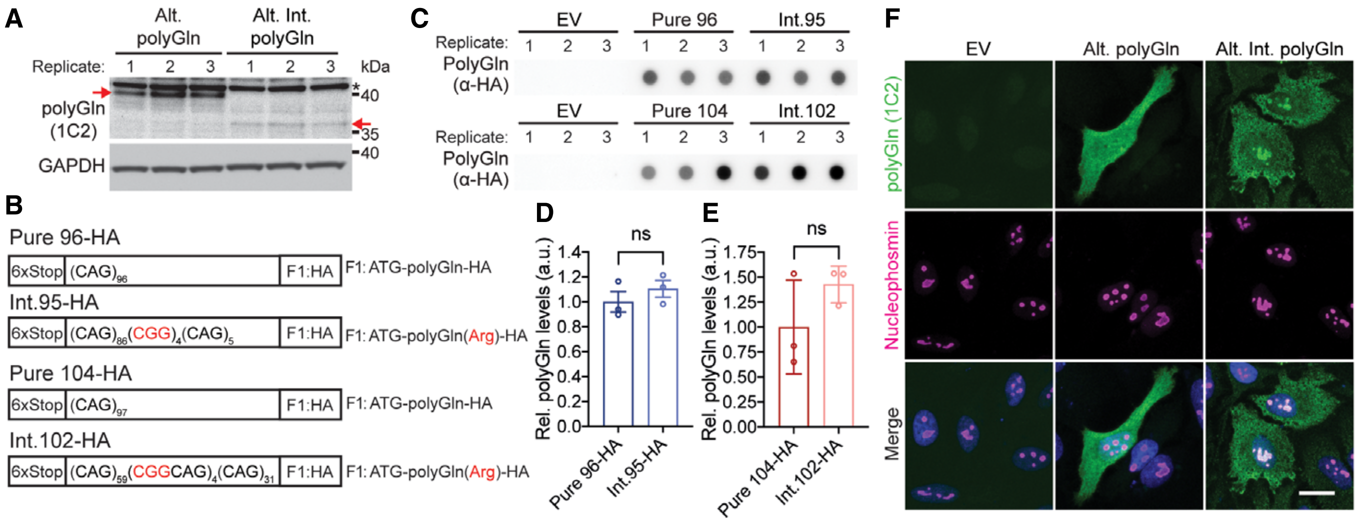

Figure EV2. Arginine interruptions alter physical properties of *ATXN8* polyGln protein.

A Protein blots of transfected HEK293T lysates show polyGln proteins expressed from constructs with interrupted or pure CAA repeat tracts; red arrows indicate pure polyGln and polyGln(Arg) proteins. * The low levels of recombinant protein expressed for toxicity studies allow for polyGln containing TATA-binding protein to be detected by 1C2 antibody giving a background band at ~ 40 kDa.

B Constructs used to express pure and interrupted HA-tagged polyGln proteins.

C–E Protein blot (C) and quantification (D, E) of insoluble protein fraction to detect polyGln proteins by HA epitope tag; EV: empty vector $n = 3$ transfections, ns: not significant, unpaired *t*-test, mean ± SEM.

F PolyGln(Arg) proteins but not pure polyGln proteins colocalize with nucleophosmin in HeLa cells; scale bars: 20 μm.

Source data are available online for this figure.

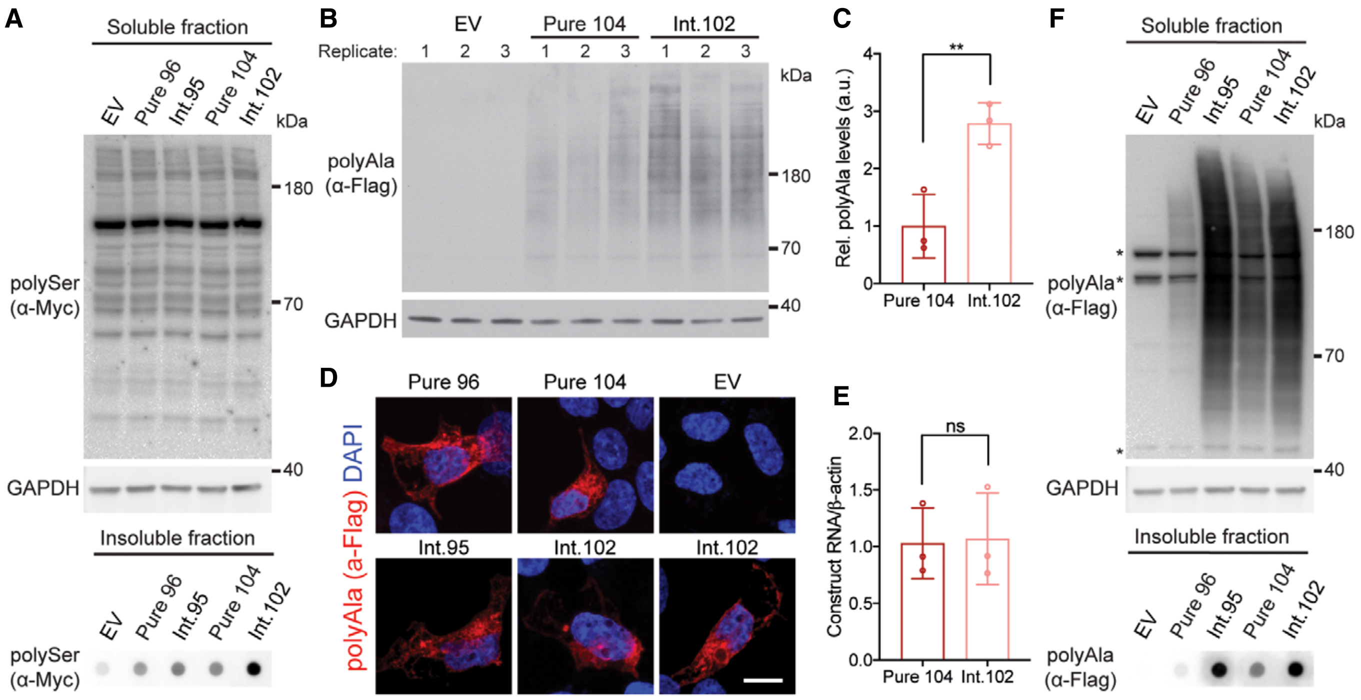

Figure EV3. CGG interruptions increase levels of RAN polyAla expansion proteins.

A Protein blot of soluble and dot blot of insoluble protein fractions for RAN polySer demonstrating that polySer RAN proteins are detectable in the insoluble fraction by dot blot but not the soluble fraction, as no signal above background is found in the soluble fractions; EV: empty vector.

B, C Protein blot (B) and quantification (C) of polyAla RAN proteins in HEK293T cells expressed from interrupted (Int.102) and pure (Pure 104) CAG repeat tracts; EV: empty vector; $n = 3$ transfections, $^{**}P < 0.01$, unpaired t-test, mean ± SD.

D Immunofluorescence of RAN polyAla proteins in HEK293T cells; scale bar 10 μm.

E RT–qPCR of transcripts expressed from Pure 104 and Int.102 constructs; $n = 3$ transfections; ns: not significant, unpaired t-test, mean ± SEM.

F Protein blots of soluble and dot blots of insoluble RAN polyAla proteins expressed from interrupted alleles show increased levels of both soluble and insoluble RAN polyAla compared with those expressed from uninterrupted repeats; * indicates background bands; EV: empty vector.

Source data are available online for this figure.

 © 2021 The Authors

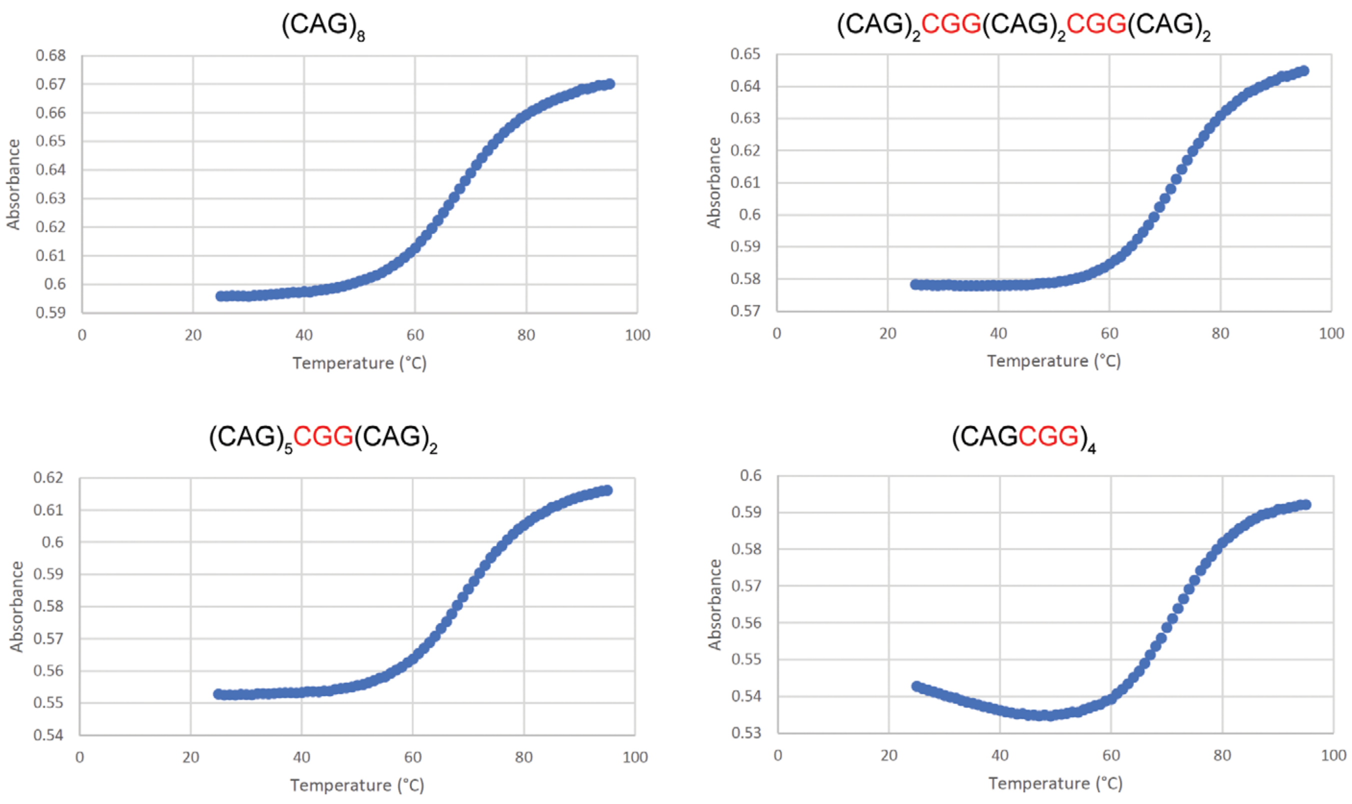

Figure EV4. CGG interruptions increase stability of CAG repeat RNA hairpins.

Example UV melting absorbance curves (for Fig 5A) for pure and interrupted RNA oligos measured at 260 nm monitored between 25 and 95°C, recorded at 1°C intervals.

Figure EV5. Predicted RNA structures for pure CAG repeat tracts and CGG-interrupted CAG repeat tracts.

A, B Predicted RNA hairpin structures from m-fold (Zuker, 2003) for pure and CGG-interrupted CAG repeat tracts for Fig 5B (A) and Fig 5C (B). Red lines alongside the structures indicate positions of CGG interruptions. (B) Pure structures are shown in gray; for repeat tracts with multiple predicted hairpin structures, only the most stable structure is shown.

© 2021 The Authors

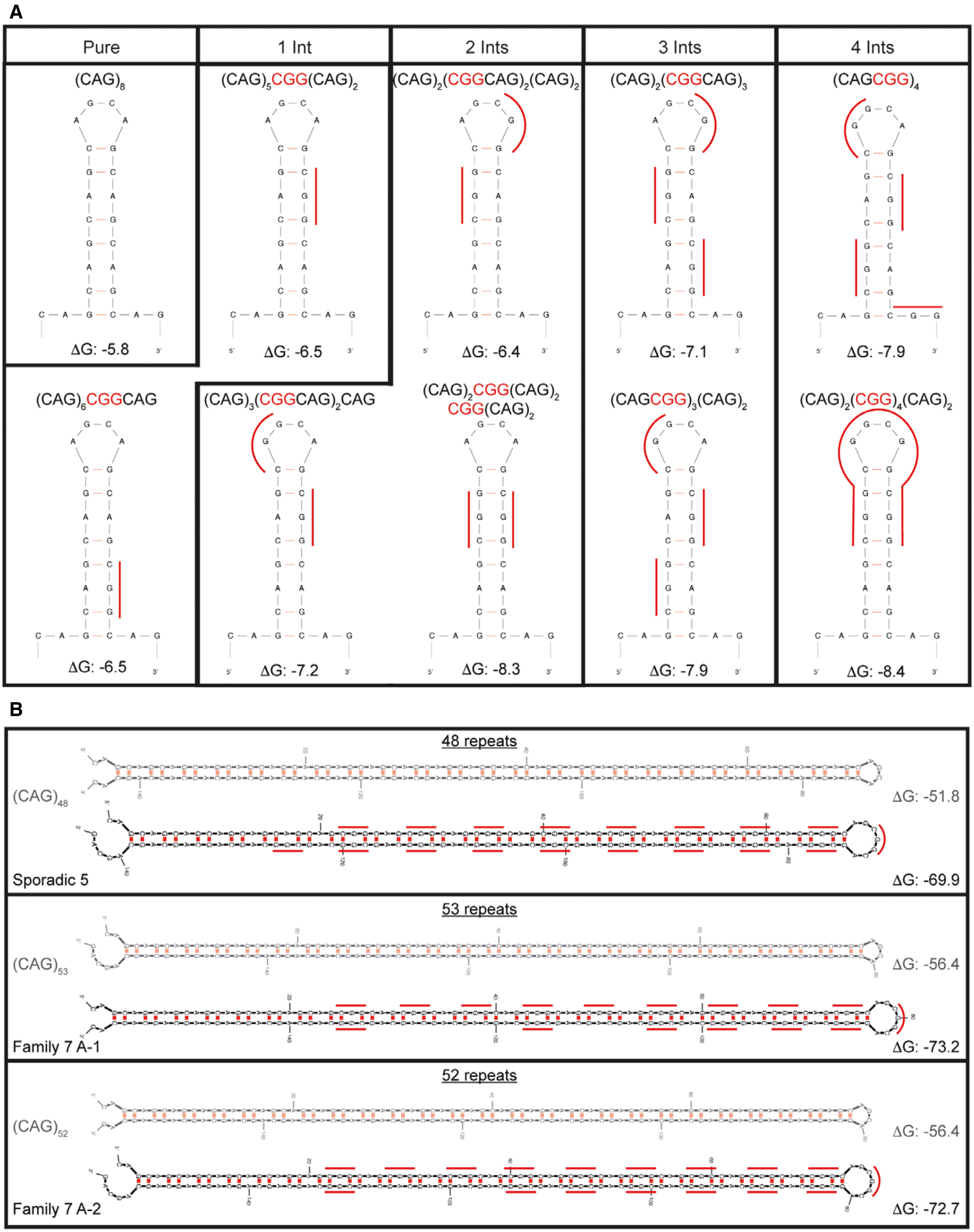

Figure EV5.

© 2021 The Authors
