# Supplementary figures and images for: CCG•CGG interruptions in high‐penetrance SCA8 families increase RAN translation and protein toxicity
Source: EMBO Mol Med. 2021 Oct 11;13(11):e14095. doi: 10.15252/emmm.202114095 (PMC8573593; doi:10.15252/emmm.202114095)

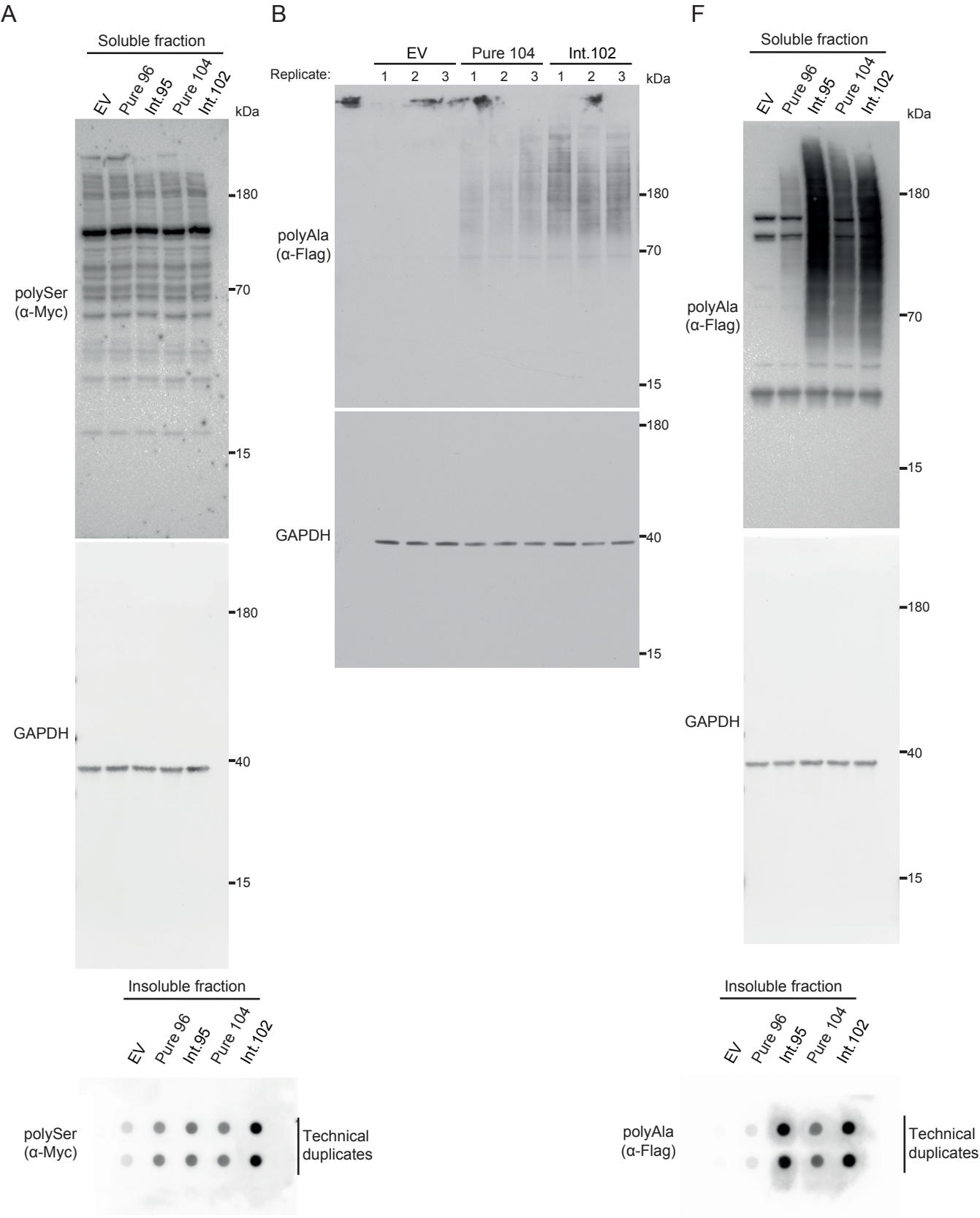

Supplement: Supplementary file 3 — Source Data for Expanded View [file EMMM-13-e14095-s005.zip › EMM-2021-14095-V3-Source_data_for_Fig EV3.pdf]

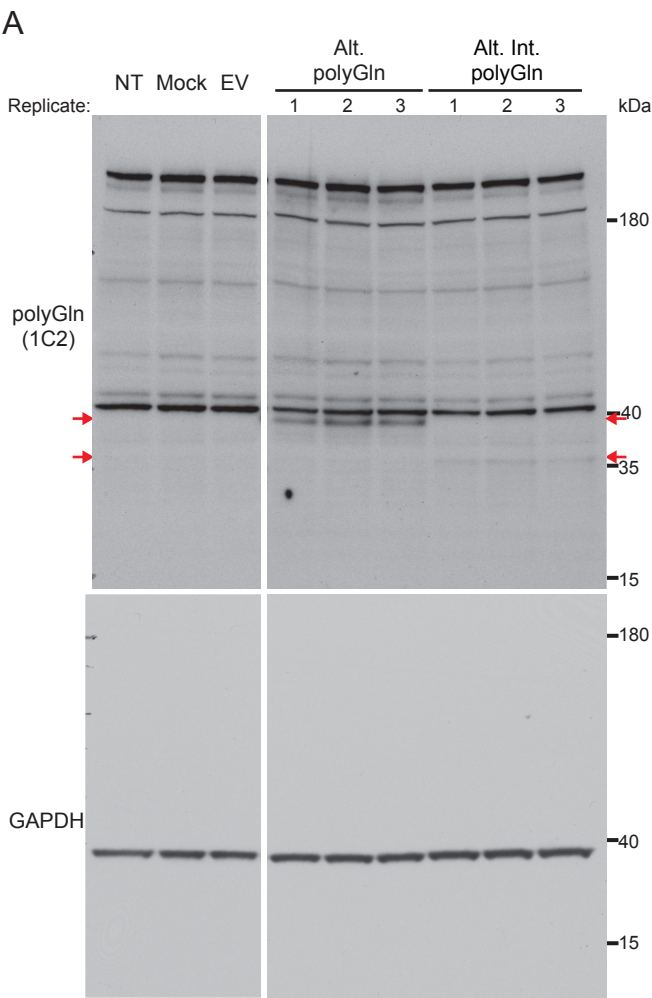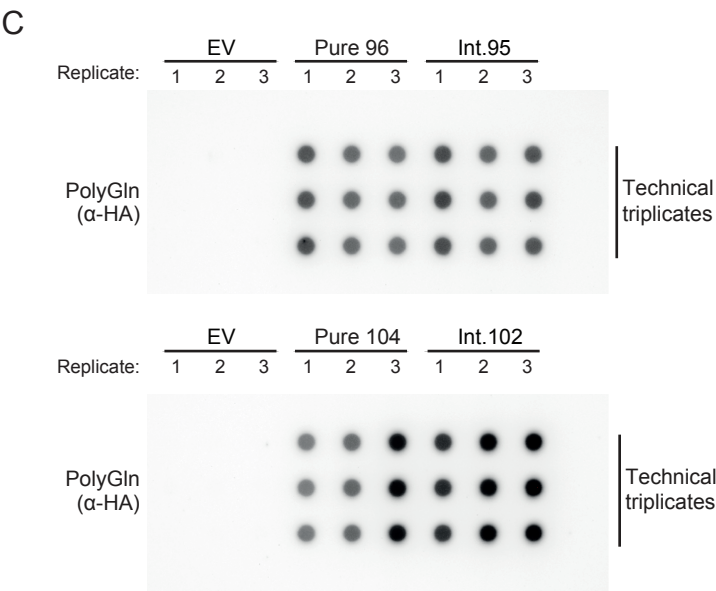

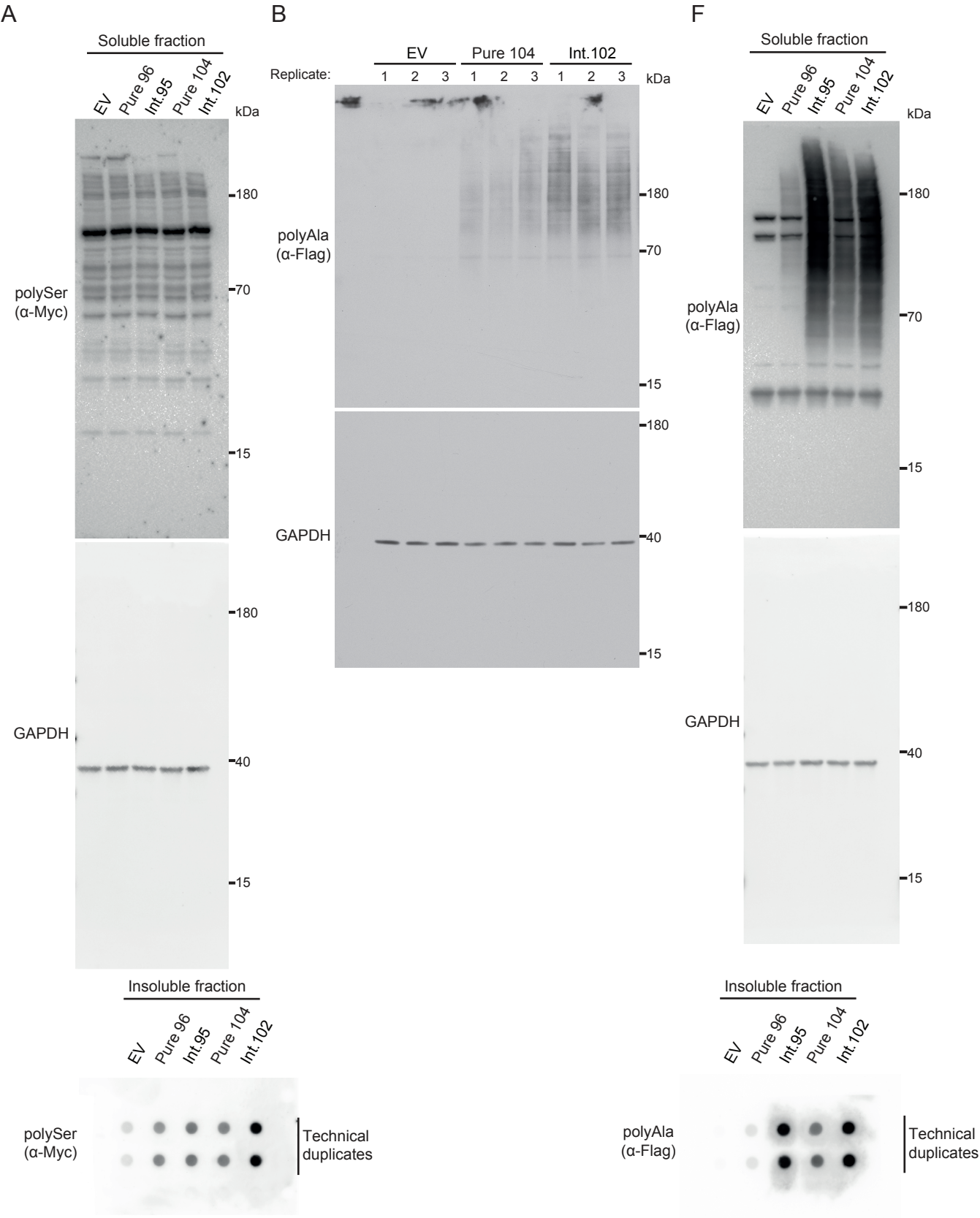

Supplement: Supplementary file 3 — Source Data for Expanded View [file EMMM-13-e14095-s005.zip › EMM-2021-14095-V3-Source_data_for_Fig EV2.pdf]

Source data: Figure 3

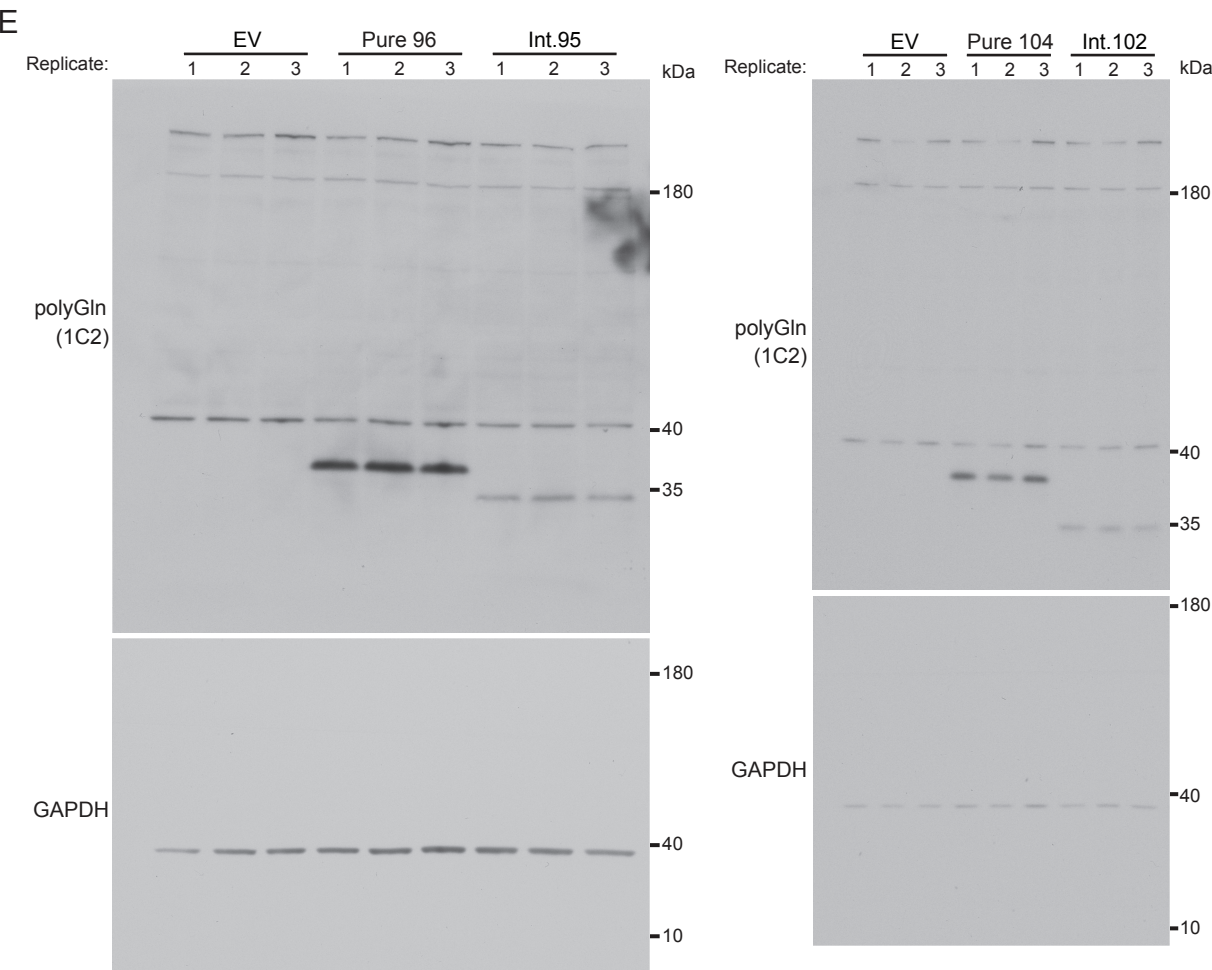

Supplement: Supplementary file 4 — Source Data for Figure 3 [file EMMM-13-e14095-s004.zip › EMM-2021-14095-V2_SourceDataForFigure3/EMM-2021-14095-V2_SourceDataForFigure3.pdf]

Source data: Figure 4

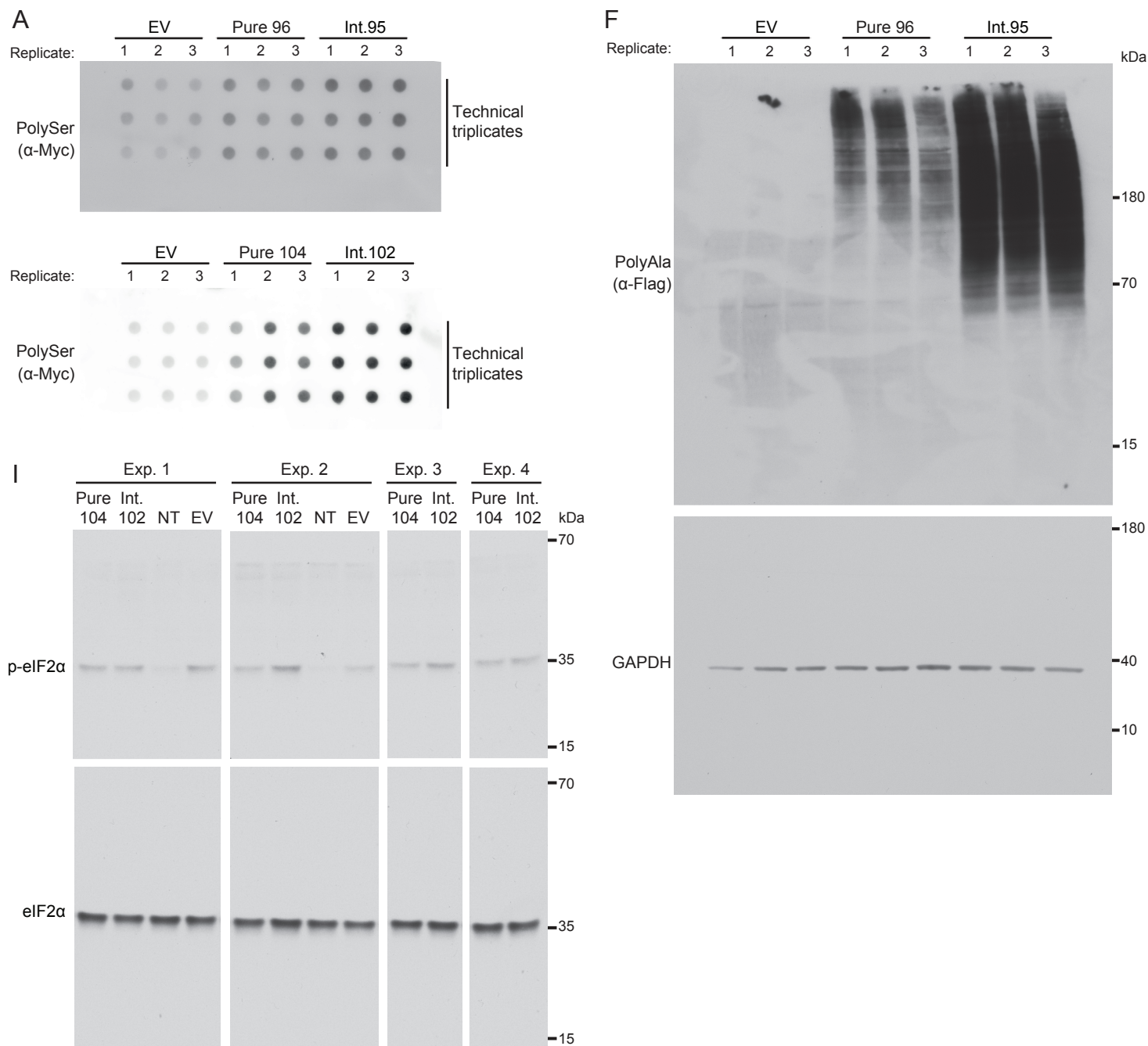

Supplement: Supplementary file 5 — Source Data for Figure 4 [file EMMM-13-e14095-s006.zip › EMM-2021-14095-V2_SourceDataForFigure4_Folder2/EMM-2021-14095-V2_SourceDataForFigure4.pdf]
