# Supplementary material for: CCG•CGG interruptions in high‐penetrance SCA8 families increase RAN translation and protein toxicity
Source: EMBO Mol Med. 2021 Oct 11;13(11):e14095. doi: 10.15252/emmm.202114095 (PMC8573593; doi:10.15252/emmm.202114095)
Supplement: Supplementary file 6 [file EMMM-13-e14095-s001.pdf]

EMBO Molecular Medicine

CCG•CGG interruptions in high penetrance SCA8 families increase RAN translation and protein toxicity

Barbara Perez, Hannah Shorrock, Monica Banez-Coronel, Tao Zu, Lisa Romano, Lauren Laboissonniere, Tammy Reid, Yoshio Ikeda, Kaalak Reddy, Christopher Gomez, Thomas Bird, Tetsuo Ashizawa, Lawrence Schut, Alfredo Brusco, Andrew Berglund, Lis Hasholt, Jorgen Nielsen, S. Subramony, and Laura Ranum
DOI: 10.15252/emmm.202114095

Corresponding author(s): Laura Ranum (ranum@ufl.edu)

Review Timeline:

Submission Date:	7th Feb 21
Editorial Decision:	8th Mar 21
Revision Received:	26th Jul 21
Editorial Decision:	20th Aug 21
Revision Received:	9th Sep 21
Accepted:	10th Sep 21

Editor: Zeljko Durdevic

Transaction Report:

(Note: With the exception of the correction of typographical or spelling errors that could be a source of ambiguity, letters and reports are not edited. Depending on transfer agreements, referee reports obtained elsewhere may or may not be included in this compilation. Referee reports are anonymous unless the Referee chooses to sign their reports.)

8th Mar 2021

Dear Dr. Ranum,

Thank you for the re-submission of your manuscript to EMBO Molecular Medicine. We have now received feedback from the three reviewers who agreed to evaluate your manuscript. As you will see from the reports below, while the referee #1 is overall supporting publication of the manuscript, referees #2 and #3 acknowledge the interest of the study but also raise serious concerns that should be addressed in a major revision. Particular attention should be given to providing more insight in the underlying mechanism of the association of repeat interruptions with increased penetrance and age at onset.

Addressing the reviewers' concerns in full will be necessary for further considering the manuscript in our journal, and acceptance of the manuscript will entail a second round of review. EMBO Molecular Medicine encourages a single round of revision only and therefore, acceptance or rejection of the manuscript will depend on the completeness of your responses included in the next, final version of the manuscript. For this reason, and to save you from any frustrations in the end, I would strongly advise against returning an incomplete revision.

We realize that the current situation is exceptional on the account of the COVID-19/SARS-CoV-2 pandemic. Therefore, please let us know if you need more than three months to revise the manuscript.

I look forward to receiving your revised manuscript.

Yours sincerely,

Zeljko Durdevic

***** Reviewer's comments *****

Referee #1 (Comments on Novelty/Model System for Author):

The work overall is of high technical quality and is of impact medically. Model system used in cells in culture which is fine.

Referee #1 (Remarks for Author):

This is a fascinating study describing one potential mechanism that underlies complexities of SCA8. In this disease, there is a high lack of penetrance of repeat expansions. The authors analyze a large number of families and pedigrees and discover that there is no relationship between age of onset and repeat length, which is quite different from most other CAG-associated diseases. In analyzing the CAG-repeat sequences, they notice from a few families (one large family in particular), that there appears to be an association of CCG repeat interruptions with penetrance. Launching from this, they use various in vitro approaches to investigate potential molecular mechanisms. By comparing proteins that are pure polyQ or with an interrupted amino acid (Arg or gly) they document that the proteins have slightly higher toxicity bearing an interrupted amino acid. They compare RAN translation of constructs of pure vs interrupted repeats for proteins produced and see that more protein is produced from constructs with an interruption vs pure repeat sequence. They also examine folding and stability of the predicted RNAs with m-fold. Their discussion is particularly thoughtful.

This is overall an interesting and well done study. Comments are largely minor, a few for additional rigor and most for clarification.

In the abstract, line 11, it would be helpful to indicate the experiments are in vitro. "At the molecular level, experiments in cells show that CCG etc.." because as a reader I was expecting that they were going to show this in human patient tissue.

In figure 1G, why the A vs AS individuals are listed in the order that they are is unclear (maybe there is a good reason). It seems clustering or grouping the A and AS individuals for each family would make the trend more clear and also make the point that this is one potential mechanism and there are likely others (which they do mention in the intro and discussion). Is it possible to asterisk in G the sequences corresponding to pg8, lines 11-20?

In Figure 3D, it is possible to perform dotblots with 1C2 as they did in figure 4A, to also assess protein level, in the advent that the protein is aggregated.

Figure 3G should be quantitated, so it is clear how representative the images shown are.
Same comment for Figure 4C.

In figure 5C, the legend is unclear. How do the patient alleles listed in the figure legend relate to the graph? And how does Figure 1G relate to that figure panel? Figure 1G is referred to in the legend.

Table 1, Pg. 34, legend. They note that additional families were sequenced "and found to carry different interruptions." I presume they mean were not found to carry CGG interruptions or not only CGG interruptions? It is a bit unclear.

In figure EV1A, the open circles are not specified. Presumably those are individuals with pure CAG repeats that have disease.

In general, I suggest a number of the data in the supplemental data be moved into the main text. They are either very nice informative figures, or essential data that the reader definitely wants to see. These include EV1, EV2, EV3A, and Table EV1.

Referee #2 (Remarks for Author):

This is a manuscript reporting that CGG interruption in SCA8 alleles explains penetrance: in highly penetrant families CGG repeat tends to present in CTG/CAG repeat expansion alleles. The authors' findings shed light on a long-term mystery why SCA8 repeat expansion shows low penetrance.

This review has some reservations.
1) Please clarify if longer CGG repeat caused stringer cell death shown in Figure 2. In LDH assay, pure Glutamine repeat caused cell death, while the cell death only slightly increased by CGG interruption. Also, how do the authors reconcile strong cell death with glutamine with low penetrance?
2) Please clarify if there were any other interruptions with different sequences (for example, TGG).
3) In Figure 3D, mobility of poly-glutamine and interrupted poly-glutamine is quite different while the length of expansions are the same. Is this what the authors expected?
4) This reviewer is wondering if the cell death induced by CGG interruption really explains penetrance. Ideally, it should be explained in gonadal cells. Does the CGG interruption make the repeat more unstable?
5) This reviewer suspects many readers would want to know how to diagnose SCA8 in respect of the repeat length, CGG interruptions and penetrance. Particularly, this work did not seem to clarify how we should consider about sporadic cases with SCA8 (ATXN8OS) CTG expansions. It may be better to show a table that classify how possible the diagnosis is with different numbers of CGG interruptions, number of affected individuals in his/her family.

Referee #3 (Comments on Novelty/Model System for Author):

Use of an animal model system in addition to cell culture would greatly improve the significance of the findings.

Referee #3 (Remarks for Author):

This manuscript investigates the cause for incomplete penetrance in SCA8, a spino-cerebellar ataxia caused by a CTG•CAG expansion in the corresponding gene. By analyzing a large cohort of families in which the disease manifests with various penetrance, the authors convincingly show that repeat interruptions, in particular interruption by CCG•CGG, positively correlate with penetrance and age at onset. In the effort to elucidate the underlying mechanism, they provide some data showing that the presence of these interruptions: 1) increase RNA hairpin stability; 2) increase RNA poly-Ala and Poly-Ser; 3) increase the toxicity of the expressed protein, carrying poly-glutamine repeats. This part appears however still rather preliminary and the experiments performed fail to convincingly reveal the underlying mechanism.

Specific comments:

1) The experiments performed to tackle the reason why repeat interruptions are associated with increased penetrance and age at onset appear at this stage still preliminary. The authors express in cells constructs with similar length of pure or interrupted repeats and use as read-out LDH and MTT assays to assess cell death and viability. Since these experiments are all performed in

overexpression, it is important to exclude difference in RNA levels, which is shown by the authors in the supplementary figures. However, in these experiments it is not clear what the individual data points in the figures 2 and 3 are. Are these independent transfections? Why there are many more experiments performed to assess viability and cell death than RNA level? Can this be the cause why some differences are significant (at $p < 0.05$) and others not?

2) Figure 3G and 4G use Hek293 cells to overexpress pure or interrupted poly-Gly or RAN poly-Ser. The authors emphasize that the expression pattern is different and this may the reason of increased toxicity, however there is no in-depth characterization of the different structures observed. What are the dotty structures in Figure 3G? What about the cytoplasmic staining that can also be observed? Is this pattern only seen in Hek293? Unfortunately, one does not learn anything about the reason for increased toxicity from these experiments.

3) The authors propose that the interruptions increase RAN translation. They show western blots or dot-blots (not clear why they use dot blots in one case) to prove this. It would be really important to show also how soluble these repeats are. The proteins in the insoluble pellets should be also shown and quantified.

We thank the reviewers for their careful comments and suggestions. We have now added considerable additional data to our manuscript and address these changes and each of the reviewer concerns below. We believe these additional data substantially improve our manuscript, which we hope is now suitable for publication in EMBO Molecular Medicine.

Referee #1 (Comments on Novelty/Model System for Author):

The work overall is of high technical quality and is of impact medically. Model system used in cells in culture which is fine.

Referee #1 (Remarks for Author):

This is a fascinating study describing one potential mechanism that underlies complexities of SCA8. In this disease, there is a high lack of penetrance of repeat expansions. The authors analyze a large number of families and pedigrees and discover that there is no relationship between age of onset and repeat length, which is quite different from most other CAG-associated diseases. In analyzing the CAG-repeat sequences, they notice from a few families (one large family in particular), that there appears to be an association of CCG repeat interruptions with penetrance. Launching from this, they use various in vitro approaches to investigate potential molecular mechanisms. By comparing proteins that are pure polyQ or with an interrupted amino acid (Arg or gly) they document that the proteins have slightly higher toxicity bearing an interrupted amino acid. They compare RAN translation of constructs of pure vs interrupted repeats for proteins produced and see that more protein is produced from constructs with an interruption vs pure repeat sequence. They also examine folding and stability of the predicted RNAs with m-fold. Their discussion is particularly thoughtful.

This is overall an interesting and well done study. Comments are largely minor, a few for additional rigor and most for clarification.

We thank the reviewer for their positive comments and helpful suggestions.

1) In the abstract, line 11, it would be helpful to indicate the experiments are in vitro. "At the molecular level, experiments in cells show that CCG etc.." because as a reader I was expecting that they were going to show this in human patient tissue.

We now state in the abstract that experiments were performed in cell culture:

> Page 2, lines 10-12: *"At the molecular level, CCG•CGG interruptions increase RNA hairpin stability and in cell culture experiments increase p-eIF2α and polyAla and polySer RAN protein levels."*

2) In figure 1G, why the A vs AS individuals are listed in the order that they are is unclear (maybe there is a good reason). It seems clustering or grouping the A and AS individuals for each family would make the trend more clear and also make the point that this is one potential mechanism and there are likely others (which they do mention in the intro and discussion). Is it possible to asterisk in G the sequences corresponding to pg8, lines 11-20?

We have now clustered the affected and asymptomatic individuals within each family in figure 1I (previously figure 1G) and have marked the sequence in the figure that corresponds to the allele described on page 7 line 13 (previously page 8 line 12) of the text with a "#" superscript.

3) In Figure 3D, it is possible to perform dotblots with 1C2 as they did in figure 4A, to also assess protein level, in the advent that the protein is aggregated.

We have now performed dot blots with the 1C2 antibody on the insoluble fraction and did not see polyGln proteins expression above the background (empty vector) levels for this antibody so we developed a second set of pure and interrupted CAG constructs where the TAG stop codons had been removed and a 3' HA epitope tag was added so that the polyGln protein could be detected using HA antibodies. We now include dot blots of insoluble

polyGln protein detected by an HA-antibody and show that there are no significant changes in the levels of pure polyGln versus interrupted polyGln(Arg) proteins in the insoluble fraction (Fig EV2B-E):

> Page 11 lines 8-10: *"Dot blots of insoluble protein fractions from HEK293T cells transfected with CAG-repeat constructs do not show significant differences in pure polyGln versus polyGln(Arg) levels (Fig EV2B-E)."*

4) Figure 3G should be quantitated, so it is clear how representative the images shown are. Same comment for Figure 4C.

We have now quantified the polySer aggregates and show that CGG interruptions increase the polySer aggregate burden per cell (Figure 4E). We also quantify polySer protein levels by dot blot in cells transfected with the Pure 102 and Int. 104 constructs (Figure 4A, C).

> Page 12, lines 14-20: *"Transfections with constructs containing interspersed CGG interruptions (Int.102) showed 85.1% higher levels of RAN polySer(Gly) compared to pure RAN polySer proteins (Pure 104) (p<0.05; Fig 4A, C). Similarly, immunofluorescence showed RAN polySer(Gly) proteins form globular or clustered aggregates compared to punctate aggregates formed by pure polySer RAN proteins (Fig 4D). Additionally, total aggregate burden is greater in cells expressing polySer(Arg) compared to pure polySer (p<0.01 for each experiment; Fig 4E)."*

We have also now quantified the number of intranuclear inclusions present in cells transfected with the alternative codon polyGln (Alt. polyGln) and alternative codon polyGln arginine interrupted (Alt. Int. polyGln) constructs (Figure 3J).

> Page 11, line 14-18: *"Similarly, in HEK293T cells transfected with CAA- or CAA/AGA-interrupted constructs, 73% of cells overexpressing polyGln(Arg) proteins have one or more intranuclear droplet-like inclusions (p<0.0001; Fig 3I, J), which are only rarely (>1%) found in cells overexpressing pure polyGln proteins (Fig 3J)."*

5) In figure 5C, the legend is unclear. How do the patient alleles listed in the figure legend relate to the graph? And how does Figure 1G relate to that figure panel? Figure 1G is referred to in the legend.

We have now added allele configurations to the X-axis labels in the legend for Figure 5C. We have also marked the sequences used in Figure 5C in Figure 1I with asterisks.

6) Table 1, Pg. 34, legend. They note that additional families were sequenced "and found to carry different interruptions." I presume they mean were not found to carry CGG interruptions or not only CGG interruptions? It is a bit unclear.

We have now updated the legend for Table 2 (previously Table 1) to specify the other types of interruptions that were seen in this study. We found one family with CGG interrupted alleles (Fig 1I Family 4) had a single GAG between the CAG expansion and the polymorphic TAG tract. An additional n=10 families, representing n=19 expansion carriers, were sequenced and found to carry different interruptions. The interruptions in these families included CTG, TAG, TGG, AAG, GAG, TAC, CCG (in the CAG direction) that were not found in combination with CGG interruptions.

7) In figure EV1A, the open circles are not specified. Presumably those are individuals with pure CAG repeats that have disease.

We now state in legend to Figure 1B (previously Figure EV1A) that open circles indicate SCA8 patients with pure CAG repeat expansions.

8) In general, I suggest a number of the data in the supplemental data be moved into the main text. They are either very nice informative figures, or essential data that the reader definitely wants to see. These include EV1, EV2, EV3A, and Table EV1.

We have incorporated Figure EV1 into Figure 1 (panels B and C), Figure EV2 into Figure 2 (panels D and E), Figure EV3A into Figure 3 (panel D) and we have moved Table EV1 to the

main manuscript as Table 1. We really appreciate these suggestions and think that they improve the flow and readability of the manuscript.

Referee #2 (Remarks for Author):

This is a manuscript reporting that CGG interruption in SCA8 alleles explains penetrance: in highly penetrant families CGG repeat tends to present in CTG/CAG repeat expansion alleles. The authors' findings shed light on a long-term mystery why SCA8 repeat expansion shows low penetrance.

This review has some reservations.
1) Please clarify if longer CGG repeat caused stringer cell death shown in Figure 2. In LDH assay, pure Glutamine repeat caused cell death, while the cell death only slightly increased by CGG interruption. Also, how do the authors reconcile strong cell death with glutamine with low penetrance?

We thank the reviewer for these comments and separately answer each question below:

1a) Please clarify if longer CGG repeat caused stringer cell death shown in Figure 2

Because the RNA levels for each repeat size-matched pair were internally controlled but there was more RNA in the shorter less viable construct set we cannot directly compare the effects of repeat length on toxicity in this experiment. While it could be interesting to compare the repeat length effects in the future, the focus of the experiments in this study was to determine the effects of repeat interruptions, because interruptions but not repeat length are correlated with age of onset and disease penetrance.

1b) In LDH assay, pure Glutamine repeat caused cell death, while the cell death only slightly increased by CGG interruption. Also, how do the authors reconcile strong cell death with glutamine with low penetrance?
We thank the reviewer for this comment and have now edited the text to address this point in the following sections:

Page 16 line 20 to page 17 line 4: *"Here we show that polyGln(Arg) interrupted proteins are more toxic than pure polyGln proteins and that steady state levels of RAN polyAla and polySer proteins expressed from interrupted repeats are increased. This increase in toxicity caused by CCG•CGG interruptions could be sufficient to tip the balance of cellular homeostasis to degeneration and cell death in cells sensitive to the SCA8 repeat expansion. Taken together, it is possible that CCG•CGG interruptions in SCA8 patients increase overall cellular toxicity and RAN protein load which may in turn exacerbate the associated pathologies, including white matter defects (Ayhan et al., 2018), in SCA8."*

Page 19, lines 3-15: *"While additional types of AT-rich sequence interruptions (e.g. CTT•AAG, CCA•TGG, CTA•TAG) have been reported in SCA8 (Hu et al., 2017; Moseley et al., 2000b), the lack of highly penetrant SCA8 families with AT-rich interruptions (Moseley et al., 2000b) makes it unlikely that they increase disease penetrance in a manner similar to CGG interruptions. This is consistent with the prediction that AT-rich interruptions decrease RNA structural stability of CAG expansion transcripts in contrast to CGGs, which increase RNA secondary structure stability. A small number of sporadic cases are homozygous for the expansions suggesting the presence of two SCA8 expansion alleles may also increase disease penetrance (Fig 1B). The fact that SCA8 is also found with reduced penetrance in patients with single uninterrupted expansion mutations suggests that, similar to other neurodegenerative diseases, trans-genetic modifiers and environmental factors are also likely to contribute to disease (Hosseinibarkooie et al, 2017; Mo et al, 2015)."*

2) Please clarify if there were any other interruptions with different sequences (for example, TGG).

We have now updated the legend for Table 2 (previously Table 1) to specify the other types of interruptions that were seen in this study. We found one family with CGG interrupted alleles (Fig 1I Family 4) had a single GAG between the CAG expansion and the polymorphic TAG tract. An additional n=10 families, representing n=19 expansion carriers, were sequenced and found to carry different interruptions. The interruptions in these families, which included CTG, TAG, TGG, AAG, GAG, TAC, CCG (in the CAG direction), were not found in combination with CGG interruptions.

3) In Figure 3D, mobility of poly-glutamine and interrupted poly-glutamine is quite different while the length of expansions are the same. Is this what the authors expected?

Based on previous observations, repetitive proteins (including polyglutamine proteins) often do not run at their expected molecular weights on SDS-PAGE gels. We find it interesting but not that surprising that the positively charged arginine interruptions change the conformation and mobility of the polar uncharged polyglutamine proteins.

> To clarify this point we have modified the text on page 11, lines 3-5: *"The change in mobility is likely caused by the introduction of positively charged arginine interruptions in these polar uncharged polyglutamine proteins."*

4) This reviewer is wondering if the cell death induced by CGG interruption really explains penetrance. Ideally, it should be explained in gonadal cells. Does the CGG interruption make the repeat more unstable?

We have now performed a transmission analysis which shows no significant difference in intergenerational repeat length changes for pure vs. CGG interrupted alleles for paternal or maternal transmission (Figure EV1).

> Page 9, lines 3-6: *"We saw no significant difference in changes in repeat length on paternal or maternal transmission of pure versus CCG•CGG interrupted alleles (paternal transmission p=0.5314, maternal transmission p=0.5748; Fig EV1)."*

5) This reviewer suspects many readers would want to know how to diagnose SCA8 in respect of the repeat length, CGG interruptions and penetrance. Particularly, this work did not seem to clarify how we should consider about sporadic cases with SCA8 (ATXN8OS) CTG expansions. It may be better to show a table that classify how possible the diagnosis is with different numbers of CGG interruptions, number of affected individuals in his/her family.

We thank the reviewer for this suggestion and have added Table 3 to clarify this point. We have also modified the discussion as follows:

> Page 16, lines 10-13: *"For asymptomatic SCA8 expansion carriers, the risk of developing ataxia is increased by the presence of CCG•CGG interruptions, which more frequently occur in families with a prior history of ataxia. However, SCA8 ataxia patients may or may not have a family history of disease or sequence interruptions (Table 3)."*

Referee #3 (Remarks for Author):

This manuscript investigates the cause for incomplete penetrance in SCA8, a spino-cerebellar ataxia caused by a CTG•CAG expansion in the corresponding gene. By analyzing a large cohort of families in which the disease manifests with various penetrance, the authors convincingly show that repeat interruptions, in particular interruption by CCG•CGG, positively correlate with penetrance and age at onset. In the effort to elucidate the underlying mechanism, they provide some data showing that the presence of these interruptions: 1) increase RNA hairpin stability; 2) increase RNA poly-Ala and Poly-Ser; 3) increase the toxicity of the expressed protein, carrying poly-glutamine repeats. This part appears however still rather preliminary and the experiments performed fail to convincingly reveal the underlying mechanism.

Specific comments:

1) The experiments performed to tackle the reason why repeat interruptions are associated with increased penetrance and age at onset appear at this stage still preliminary. The authors express in cells constructs with similar length of pure or interrupted repeats and use as read-out LDH and MTT assays to assess cell death and viability. Since these experiments are all performed in overexpression, it is important to exclude difference in RNA levels, which is shown by the authors in the supplementary figures. However, in these experiments it is not clear what the individual data points in the figures 2 and 3 are. Are these independent transfections? Why there are many more experiments performed to assess viability and cell death than RNA level? Can this be the cause why some differences are significant (at $p < 0.05$) and others not?

We thank the reviewer for these comments and have performed additional qPCR experiments on transfected cells used for the toxicity studies. These data show a trend towards lower levels of RNA for the Int.95 construct vs Pure 96 and significantly lower levels of Int.102 compared to Pure 104 (Fig 2D, E). These data demonstrate that the increased cell toxicity and the reduced cell viability that we see with the interrupted constructs are not caused by increased RNA levels.

> These data are summarized on page 9, line 23 to page 10, line 5: *"Cells expressing these interrupted constructs showed increased death (26.9%, p<0.05 – Int.95 vs Pure 96; 23.5%, p<0.05 – Int.102 vs Pure 104; Fig 2B) and decreased viability (16.5%, p<0.05 – Int.95 vs Pure 96; 15.6%, p<0.05 – Int.102 vs Pure 104; Fig. 2C) compared to length-matched uninterrupted repeats. These effects cannot be explained by differences in RNA levels which did not differ in Pure 96 versus Int.95 transfected cells and were lower in Int.102 vs Pure 104 transfected cells (Fig 2D, E)."*

Additionally, we now clarify in the methods section that MTT, LDH and RNA analyses were performed from a minimum of six independent transfections with each n representing a single independent transfection; for LDH and MTT assays, each independent transfection was performed in quintuplet.

> Page 23, lines 5-7: *"Toxicity and viability assays were performed in a minimum of six independent experiments and in each independent experiment the assays were performed in quintuplet."*

2) Figure 3G and 4G use Hek293 cells to overexpress pure or interrupted poly-Gly or RAN poly-Ser. The authors emphasize that the expression pattern is different and this may the reason of increased toxicity, however there is no in-depth characterization of the different structures observed. What are the dotty structures in Figure 3G? What about the cytoplasmic staining that can also be observed? Is this pattern only seen in Hek293? Unfortunately, one does not learn anything about the reason for increased toxicity from these experiments.

We have performed a number of additional experiments to better understand how the interruptions increase toxicity. We show that the interruptions increase the integrated stress response, which has been previously shown to increase RAN translation and have also better characterized the repeat protein pathology and localization in multiple cell types.

First, we show that interrupted repeats increase the integrated stress response in transfected cells as described and discussed below.

> Page 13, lines 7-9: *"Additionally, we show in transiently transfected HEK293T cells that overexpression of interrupted repeats activated the integrated stress response (ISR) and increased p-eIF2α levels by 49% compared to pure repeats (p<0.05, Fig 4I, J)."*

> Page 15, lines 12-20: *"This observation is consistent with the increased stability of RNA structures predicted on CGG interrupted alleles and the elevated p-eIF2α levels. It will be interesting in future work to test if the elevated p-eIF2α levels are caused by PKR activation, which can be activated by structured microsatellite RNAs*

(Edery et al, 1989; Tian et al, 2000; Zu et al, 2020) and which has been recently shown to be a major driver of RAN translation (Zu et al., 2020) or result from feedforward effects in which the RAN proteins activate the integrated stress response and p-eIF2α, which further increases RAN translation (Cheng et al, 2018; Green et al, 2017; Sonobe et al, 2018; Tusi et al, 2021; Zu et al., 2020).”

Second, we now show that CGG interruptions increase the polySer aggregate burden (Figure 4E).

Page 12, lines 16-20: *“Similarly, immunofluorescence showed RAN polySer(Gly) proteins form globular or clustered aggregates compared to punctate aggregates formed by pure polySer RAN proteins (Fig 4D). Additionally, total aggregate burden is greater in cells expressing polySer(Arg) compared to pure polySer ($p<0.01$ for each experiment; Fig 4E).”*

Third, we have quantified the number of intranuclear inclusions (Figure 3J) and demonstrate that the polyGln(Arg) interrupted proteins colocalize with the nucleoli in both HEK293T and HeLa cells. We have revised the text to describe these new data:

Page 11, lines 14-20: *“Similarly, in HEK293T cells transfected with CAA- or CAA/AGA-interrupted constructs, 73% of cells overexpressing polyGln(Arg) proteins have one or more intranuclear droplet-like inclusions ($p<0.0001$; Fig 3I, J), which are only rarely (>1%) found in cells overexpressing pure polyGln proteins (Fig 3J). These inclusions, which are found in transfected HEK293T and HeLa cells, colocalize with the nucleolar marker nucleophosmin (Fig 3K, EV2F).”*

Fourth, we discuss these new polyGln(Arg) data and to put them into context with data from other repeat expansion disorders, we have added the following paragraph to the Discussion:

Page 17, line 15 to Page 18, line 13: *“RAN translation has now been described in 11 microsatellite expansion diseases and in several of these diseases the differences between RAN proteins that contain arginine residues and those that do not have been characterized. For example, in myotonic dystrophy type 2 (DM2), RAN translation across the sense CCTG repeat produces polyLPAC proteins which show a diffuse cytoplasmic distribution in HEK293T cells but RAN translation across the antisense CAGG repeat produces polyQAGR proteins which form droplet-like nuclear staining not found with polyLPAC proteins (Zu et al., 2017). Similarly, in C9orf72 ALS/FTD, polyGR and polyPR form droplet-like nuclear structures that colocalize with nucleolar markers while polyGA, polyGP and polyPA show diffuse nuclear and cytoplasmic localization (Tao et al, 2015; Wen et al, 2014). Furthermore, the arginine containing polyGR and polyPR proteins are more toxic in cell culture and Drosophila than the dipeptide repeat proteins that do not contain arginine residues (Lee et al, 2016; Mizielinska et al, 2014; Tao et al., 2015; Wen et al., 2014) and they have been shown to impair ribosomal RNA biogenesis, perturb stress granule dynamics and biomolecular phase separation, and induce nucleolar stress (Boeynaems et al, 2017; Kwon et al, 2014; Lee et al., 2016; Tao et al., 2015; White et al, 2019). Similar to the polyGR and polyPR proteins found in C9orf72 ALS/FTD, the SCA8 arginine-interrupted polyGln(Arg) proteins show increased toxicity and droplet-like nucleolar inclusions. These striking molecular parallels between SCA8, DM2 and C9orf72 ALS/FTD suggest similar molecular mechanisms are at play and that the effects of polyGln(Arg) proteins on nucleolar stress and stress granule dynamics warrant further investigation.”*

3) The authors propose that the interruptions increase RAN translation. They show western blots or dot-blots (not clear why they use dot blots in one case) to prove this. It would be really important to show also how soluble these repeats are. The proteins in the insoluble pellets should be also shown and quantified.

We have now included matched Western and dot blots of soluble and insoluble fractions for RAN polySer (Figure EV3A) and RAN polyAla (Figure EV3F) and have modified the text as follows:

> Page 12, lines 9-14: *"In the polySer reading frame, the GGC interruptions produce a polySer protein with glycine interruptions, polySer(Gly). Both pure polySer and polySer(Gly) proteins are highly insoluble with no protein detected in the soluble fraction (Fig EV3A). Dot blot analyses of the insoluble protein fraction showed 93.8% higher levels of interrupted RAN polySer(Gly) compared to pure RAN polySer proteins (p<0.01; Fig 4A, B)."*
>
> Page 13, line 2-4. *"The increases in polyAla protein levels did not result in overt changes in cellular localization (Fig EV3D) and were found in both soluble and insoluble protein fractions (Fig EV3F)."*

20th Aug 2021

Dear Dr. Ranum,

please disragard the previous e-mail and accept my appologies for this oversight. Below is the decision letter.

Thank you for the submission of your manuscript to EMBO Molecular Medicine. I am pleased to inform you that we will be able to accept your manuscript pending the following final amendments:

1) In the main manuscript file, please do the following:
- Correct/answer the track changes suggested by our data editors by working from the attached document.
- Make sure that all special characters display well.
- Add author contributions for Thomas Bird, Alfredo Brusco and Andrew Berglund.
- Include a statement that informed consent was obtained from all human subjects and that, in addition to the WMA Declaration of Helsinki , the experiments conformed to the principles set out in the Department of Health and Human Services Belmont Report.
- In M&M, a statistical paragraph should reflect all information that you have filled in the Authors Checklist, especially regarding randomization, blinding, replication.
2) Appendix: Please put the data in the table form, name them Appendix Table S1 etc. and add table of content.
3) Figures: Remove nomenclature (A) from figures EV1 and EV4.
4) Source data: Please upload one file per figure.
5) Synopsis: Please check your synopsis text and image, revise them if necessary and submit their final versions with your revised manuscript. Please be aware that in the proof stage minor corrections only are allowed (e.g., typos).
6) For more information: There is space at the end of each article to list relevant web links for further consultation by our readers. Could you identify some relevant ones and provide such information as well? Some examples are patient associations, relevant databases, OMIM/proteins/genes links, author's websites, etc...
7) As part of the EMBO Publications transparent editorial process initiative (see our Editorial at http://embomolmed.embopress.org/content/2/9/329), EMBO Molecular Medicine will publish online a Review Process File (RPF) to accompany accepted manuscripts. This file will be published in conjunction with your paper and will include the anonymous referee reports, your point-by-point response and all pertinent correspondence relating to the manuscript. Let us know whether you agree with the publication of the RPF and as here, if you want to remove or not any figures from it prior to publication. Please note that the Authors checklist will be published at the end of the RPF.
8) Please provide a point-by-point letter INCLUDING my comments as well as the reviewer's reports and your detailed responses (as Word file).

I look forward to seeing a revised form of your manuscript as soon as possible. Use this link to login to the manuscript system and submit your revision: https://embomolmed.msubmit.net/cgi-bin/main.plex

. **** Reviewer's comments *****

Referee #2 (Remarks for Author):

Thank you very much for revising the manuscript.

Referee #3 (Remarks for Author):

The authors have substantially revised the manuscript and addressed raised concerns.

10th Sep 2021

Dear Dr. Ranum,

We are pleased to inform you that your manuscript is accepted for publication and is now being sent to our publisher to be included in the next available issue of EMBO Molecular Medicine.

EMBO PRESS

YOU MUST COMPLETE ALL CELLS WITH A PINK BACKGROUND

PLEASE NOTE THAT THIS CHECKLIST WILL BE PUBLISHED ALONGSIDE YOUR PAPER

Corresponding Author Name: Laura Ranum, PhD

Journal Submitted to: EMBO Molecular Medicine
Manuscript Number: EMM-2021-14095-V2

Reporting Checklist For Life Sciences Articles (Rev. June 2017)

This checklist is used to ensure good reporting standards and to improve the reproducibility of published results. These guidelines are consistent with the Principles and Guidelines for Reporting Preclinical Research issued by the NIH in 2014. Please follow the journal's authorship guidelines in preparing your manuscript.

A- Figures
1. Data
The data shown in figures should satisfy the following conditions:

- the data were obtained and processed according to the field's best practice and are presented to reflect the results of the experiments in an accurate and unbiased manner.
- figure panels include only data points, measurements or observations that can be compared to each other in a scientifically meaningful way.
- graphs include clearly labeled error bars for independent experiments and sample sizes. Unless justified, error bars should not be shown for technical replicates.
- if n< 5, the individual data points from each experiment should be plotted and any statistical test employed should be justified
- Source Data should be included to report the data underlying graphs. Please follow the guidelines set out in the author ship guidelines on Data Presentation.

2. Captions
Each figure caption should contain the following information, for each panel where they are relevant:

- a specification of the experimental system investigated (eg cell line, species name).
- the assay(s) and method(s) used to carry out the reported observations and measurements
- an explicit mention of the biological and chemical entity(ies) that are being measured.
- an explicit mention of the biological and chemical entity(ies) that are altered/varied/perturbed in a controlled manner.

- the exact sample size (n) for each experimental group/condition, given as a number, not a range;
- a description of the sample collection allowing the reader to understand whether the samples represent technical or biological replicates (including how many animals, litters, cultures, etc.).
- a statement of how many times the experiment shown was independently replicated in the laboratory.
- definitions of statistical methods and measures:
  - common tests, such as t-test (please specify whether paired vs. unpaired), simple χ2 tests, Wilcoxon and Mann-Whitney tests, can be unambiguously identified by name only, but more complex techniques should be described in the methods section;
  - are tests one-sided or two-sided?
  - are there adjustments for multiple comparisons?
  - exact statistical test results, e.g., P values = x but not P values < x;
  - definition of 'center values' as median or average;
  - definition of error bars as s.d. or s.e.m.

Any descriptions too long for the figure legend should be included in the methods section and/or with the source data.

In the pink boxes below, please ensure that the answers to the following questions are reported in the manuscript itself. Every question should be answered. If the question is not relevant to your research, please write NA (non applicable). We encourage you to include a specific subsection in the methods section for statistics, reagents, animal models and human subjects.

B- Statistics and general methods

Please fill out these boxes (Do not worry if you cannot see all your text once you press return)

1.a. How was the sample size chosen to ensure adequate power to detect a pre-specified effect size?	SCA8 families were included in our study if the repeat length and gender of the proband were known. Sample sizes for cell based work were chosen based on prior experience. All cell culture experiments were performed with a minimum of 3 biological replicates.
1.b. For animal studies, include a statement about sample size estimate even if no statistical methods were used.	N/A
2. Describe inclusion/exclusion criteria if samples or animals were excluded from the analysis. Were the criteria pre-established?	Patients for whom repeat size could not be determined or DNA was not available were not included in the study.
3. Were any steps taken to minimize the effects of subjective bias when allocating animals/samples to treatment (e.g. randomization procedure)? If yes, please describe.	Not applicable as samples were not allocated to groups.
For animal studies, include a statement about randomization even if no randomization was used.	Not applicable
4.a. Were any steps taken to minimize the effects of subjective bias during group allocation or/and when assessing results (e.g. blinding of the investigator)? If yes please describe.	Investigators were blinded for all quantification of immunofluorescent data.
4.b. For animal studies, include a statement about blinding even if no blinding was done	Not applicable
5. For every figure, are statistical tests justified as appropriate?	Appropriate statistical analyses were used throughout the manuscript and described in the materials and methods section and listed in the figure legends.
Do the data meet the assumptions of the tests (e.g., normal distribution)? Describe any methods used to assess it.	The D'Agostino and Pearson normality test or Sharpio-Wilk normality test were used to assess normal distribution and statistical tests for which the data meet the assumptions of the tests were selected.
Is there an estimate of variation within each group of data?	There is variability in the patient cohort which is outlined in Table 1 and Figure 1. The cell based systems are consitent across multiple transfections.

USEFUL LINKS FOR COMPLETING THIS FORM

http://www.antibodypedia.com
http://1degreebio.org
http://www.equator-network.org/reporting-guidelines/improving-bioscience-research-repor

http://grants.nih.gov/grants/olaw/olaw.htm
http://www.mrc.ac.uk/Ourresearch/Ethicsresearchguidance/Useofanimals/index.htm
http://ClinicalTrials.gov
http://www.consort-statement.org
http://www.consort-statement.org/checklists/view/32-consort/66-title
http://www.equator-network.org/reporting-guidelines/reporting-recommendations-for-tumo
http://datadryad.org
http://figshare.com
http://www.ncbi.nlm.nih.gov/gap
http://www.ebi.ac.uk/ega
http://biomodels.net/
http://biomodels.net/miriam/
http://jjj.biochem.sun.ac.za
https://osp.od.nih.gov/biosafety-biosecurity-and-emerging-biotechnology/
http://www.selectagents.gov/

Is the variance similar between the groups that are being statistically compared?	The variance is similar between all groups analysed using an unpaired two-tailed t test or a two-way ANOVA. The Mann-Whitney test was used to analyze non-parametric data where the variance differed between the groups.

C- Reagents

6. To show that antibodies were profiled for use in the system under study (assay and species), provide a citation, catalog number and/or clone number, supplementary information or reference to an antibody validation profile. e.g., Antibodypedia (see link list at top right), 1DegreeBio (see link list at top right).	All catalog numbers of antibodies are included in the materials and methods section.
7. Identify the source of cell lines and report if they were recently authenticated (e.g., by STR profiling) and tested for mycoplasma contamination.	Previously established and characterized cell lines (HEK293T, HeLa and T98) are used in this study, these were obtained from the American Type Culture Collection.

* for all hyperlinks, please see the table at the top right of the document

D- Animal Models

8. Report species, strain, gender, age of animals and genetic modification status where applicable. Please detail housing and husbandry conditions and the source of animals.	N/A
9. For experiments involving live vertebrates, include a statement of compliance with ethical regulations and identify the committee(s) approving the experiments.	N/A
10. We recommend consulting the ARRIVE guidelines (see link list at top right) (PLoS Biol. 8(6), e1000412, 2010) to ensure that other relevant aspects of animal studies are adequately reported. See author guidelines, under 'Reporting Guidelines'. See also: NIH (see link list at top right) and MRC (see link list at top right) recommendations. Please confirm compliance.	N/A

E- Human Subjects

11. Identify the committee(s) approving the study protocol.	The appropriate Institutional Review Boards approved the collection and analysis of patient DNA samples.
12. Include a statement confirming that informed consent was obtained from all subjects and that the experiments conformed to the principles set out in the WMA Declaration of Helsinki and the Department of Health and Human Services Belmont Report.	Informed consent was obtained from all participants and the experiments conformed to the principles set out in the WMA Declaration of Helsinki and the Department of Health and Human Services Belmont Report.
13. For publication of patient photos, include a statement confirming that consent to publish was obtained.	N/A
14. Report any restrictions on the availability (and/or on the use) of human data or samples.	All CAG expansion allele configurations are available in the manuscript appendix.
15. Report the clinical trial registration number (at ClinicalTrials.gov or equivalent), where applicable.	N/A
16. For phase II and III randomized controlled trials, please refer to the CONSORT flow diagram (see link list at top right) and submit the CONSORT checklist (see link list at top right) with your submission. See author guidelines, under 'Reporting Guidelines'. Please confirm you have submitted this list.	N/A
17. For tumor marker prognostic studies, we recommend that you follow the REMARK reporting guidelines (see link list at top right). See author guidelines, under 'Reporting Guidelines'. Please confirm you have followed these guidelines.	N/A

F- Data Accessibility

18: Provide a "Data Availability" section at the end of the Materials & Methods, listing the accession codes for data generated in this study and deposited in a public database (e.g. RNA-Seq data: Gene Expression Omnibus GSE39462, Proteomics data: PRIDE PXD000208 etc.) Please refer to our author guidelines for 'Data Deposition'.<br><br>Data deposition in a public repository is mandatory for:<br>a. Protein, DNA and RNA sequences<br>b. Macromolecular structures<br>c. Crystallographic data for small molecules<br>d. Functional genomics data<br>e. Proteomics and molecular interactions	N/A
19. Deposition is strongly recommended for any datasets that are central and integral to the study; please consider the journal's data policy. If no structured public repository exists for a given data type, we encourage the provision of datasets in the manuscript as a Supplementary Document (see author guidelines under 'Expanded View' or in unstructured repositories such as Dryad (see link list at top right) or Figshare (see link list at top right).	N/A
20. Access to human clinical and genomic datasets should be provided with as few restrictions as possible while respecting ethical obligations to the patients and relevant medical and legal issues. If practically possible and compatible with the individual consent agreement used in the study, such data should be deposited in one of the major public access-controlled repositories such as dbGAP (see link list at top right) or EGA (see link list at top right).	N/A
21. Computational models that are central and integral to a study should be shared without restrictions and provided in a machine-readable form. The relevant accession numbers or links should be provided. When possible, standardized format (SBML, CellML) should be used instead of scripts (e.g. MATLAB). Authors are strongly encouraged to follow the MIRIAM guidelines (see link list at top right) and deposit their model in a public database such as Biomodels (see link list at top right) or JWS Online (see link list at top right). If computer source code is provided with the paper, it should be deposited in a public repository or included in supplementary information.	N/A

G- Dual use research of concern

22. Could your study fall under dual use research restrictions? Please check biosecurity documents (see link list at top right) and list of select agents and toxins (APHIS/CDC) (see link list at top right). According to our biosecurity guidelines, provide a statement only if it could.	N/A
